# Invitation Cards during Pregnancy Enhance Male Partner Involvement in Prevention of Mother to Child Transmission (PMTCT) of Human Immunodeficiency Virus (HIV) in Blantyre, Malawi: A Randomized Controlled Open Label Trial

**DOI:** 10.1371/journal.pone.0119273

**Published:** 2015-03-03

**Authors:** Alinane Linda Nyondo, Augustine Talumba Choko, Angela Faith Chimwaza, Adamson Sinjani Muula

**Affiliations:** 1 School of Public Health and Family Medicine, College of Medicine, University of Malawi, Blantyre, Malawi; 2 Malawi Liverpool Wellcome Trust Clinical Research Programme, Blantyre, Malawi; 3 Kamuzu College of Nursing, University of Malawi, Blantyre, Malawi; Population Council, INDIA

## Abstract

**Introduction:**

Male involvement (MI) is vital for the uptake of Prevention of Mother to Child Transmission (PMTCT) of Human Immunodeficiency Virus (HIV) interventions. Partner notification (PN) is among the strategies identified for MI in PMTCT services. The purpose of this randomized controlled trial was to evaluate the efficacy of an invitation card to the male partners as a strategy for MI in PMTCT services by comparing the proportion of pregnant women that were accompanied by their partners between the intervention and the non-intervention study groups.

**Methods:**

Pregnant women attending antenatal care without a male partner at South Lunzu and Mpemba health centres in Blantyre, Malawi, were enrolled in the study from June to December 2013. In an intention-to-treat analysis, we compared all participants that were randomized in the invitation card group with the standard of care (SoC) group. Risk ratios (RR) with 95% confidence intervals (CI) were computed to assess the efficacy of the invitation card.

**Results:**

Of the 462 randomized women, 65/230 (28.26%) of the women in the invitation card group reported to the antenatal care clinic with their partners compared to 44/232 (18.97%) women in the SoC group. In an unadjusted intention-to-treat analysis women in the invitation card group were 50% more likely to be accompanied by their male partners than those in the SoC group RR: 1.49 (95% CI: 1.06-2.09); p = 0.02. Our random effects analysis showed that there was no clustering by site of recruitment with an inter cluster correlation coefficient (ICC) of 1.98x 10^-3^, (95% CI: 1.78 x10^-7^ - 0.96 x 10^-1^); p =0.403.

**Conclusion:**

An invitation card significantly increased the proportion of women who were accompanied by their male partners for the PMTCT services. An invitation card is a feasible strategy for MI in PMTCT.

## Introduction

Prevention of Mother to Child Transmission (PMTCT) of Human Immunodeficiency Virus (HIV) services remains the major way of reducing paediatric HIV infection and promoting maternal health [1]. Although there is reduction of mother to child transmission (MTCT) of HIV globally with transmission rates of <5% secondary to the comprehensive approach with PMTCT services [2], the uptake of PMTCT interventions in Sub-Saharan Africa has been suboptimal [3]. Despite the high acceptance with HIV testing among pregnant women [3] the mean coverage of antiretroviral (ARVs) prophylaxis for PMTCT was 65% in 2012 and ranged from 57%-70% and was below the global target of providing ARVs to 90% of HIV infected pregnant women [4]. Furthermore, the proportion of women who received ARVs for their own health was lower in 2012 compared to the adult proportion. Globally, the pace of reduction in the decline of primary new HIV infections in women has slowed down [4].

Increased uptake and retention of PMTCT interventions reduce MTCT of HIV and may reduce transmission between sero-discordant partners [5]. A social challenge with a woman’s uptake of PMTCT interventions is the lack of support from her male partner [6]. Male Involvement (MI) as a family-centred approach to PMTCT is neglected in the PMTCT service and deemed challenging to implement [7]. Although MI is vital for the uptake and scaling up of PMTCT interventions [7–14], reduction in MTCT [8] and uptake of maternal health services [15–18], it has remained low in Sub Saharan Africa (SSA) between 3.2%–25% [9,19–21].

Partner notification (PN) is among the strategies identified for MI in PMTCT services [22–27]. Partner notification is used for tracing and treatment of partners of clients diagnosed with sexually transmitted infections (STIs) [28,29]. Studies in Malawi showed that provider-assisted partner notification was effective and feasible among STI clinic patients [30,31] and improved linkage to care [31]; however, its effectiveness on men’s attendance to PMTCT of HIV services has not been explored [30]. Programmatic data on PMTCT services in Zambia and Uganda reported effectiveness of partner notification slips in an antenatal clinic with most men reporting to the clinic [32]. Despite the availability of MI strategies there is limited evidence of implementation of effective and consistent strategies for MI in PMTCT [9,31,33,34]. Furthermore, there are limited rigorously conducted studies that have investigated the effectiveness and feasibility of the suggested strategies. The purpose of this randomized controlled open label trial was to investigate the efficacy and feasibility of an invitation card as a strategy for MI PMTCT services by comparing the proportion of pregnant women that were accompanied by their partners at week 2 and week 6 of the study following receipt of an invitation card in the intervention and the standard of care (SoC) study groups.

## Methods

### Study Design

This was a randomized, open label controlled trial among pregnant women in the antenatal clinics to determine the feasibility and efficacy of an invitation card for MI in PMTCT services. Pregnant women were enrolled at any time point in pregnancy, until 30 weeks gestation. There were two study groups: Group A was the intervention group where women were randomized to use of an invitation card (S1 Invitation Card) as a strategy for MI in PMTCT of HIV and Group B was the SoC group where women used word of mouth invites. The message on the invitation card was similar to the word of mouth invite.

### Study Setting

The trial was conducted in Blantyre district in the southern region of Malawi. Blantyre has a population of 1,001, 984 consisting of 501,000 males and 500, 984 females as of 2008 and comprises urban, semi urban and rural areas [35]. The district has 32 health centres and all provide PMTCT services. The HIV prevalence among pregnant women in Blantyre in 2011 was 10.4% [36]. The specific health centres used were South Lunzu Health Centre which is in the North Eastern side and Mpemba Health Centre, which is in the South Eastern side of Blantyre district. The selection of the health centres was based on the different geographical areas they are located in. The health centres serve semi urban populations and offers the following services out patient, HIV Counselling and Testing, Family Planning, Directly Observed Treatment for Tuberculosis, Maternity services which have Antenatal care, Labour and delivery and Postpartum services.

### Study Population

The study population comprised all pregnant women who attended antenatal care services at South Lunzu and Mpemba health centres. Pregnant women were eligible for enrolment if they were aged 16 years or older and married, attended antenatal care without a spouse, planned to attend antenatal care at the study clinic, were able and willing to provide informed consent before participation in the study, and had a male partner who was known to be responsible for the current pregnancy, willing to pass on the “invitation card” to the partner and were below or equal to 30 weeks gestation (ascertained through fundal height assessments and or as calculated from the Last Normal Menstrual Period [LNMP] date). We limited gestation period to 30 weeks to allow for adequate evaluation of the strategy during follow up visits by minimising loss to follow up secondary to delivery.

### Sample Size

The primary outcome was the proportion of pregnant women who reported to the study clinic with their partners, for PMTCT services following use of an invitation card after 2 visits. The estimated uptake of a strategy for MI in PMTCT was 14% without intervention based on a Ugandan study [37]. In the present study, we expected to observe an increase of MI in PMTCT services from 2% (without intervention) to 12% (with intervention). A total sample size of 462 pregnant women provided 90% power at a 5% level of significance to detect a 10% increase in MI in the group that used the invitation card.

### Study Intervention

The Principal Investigator developed the invitation card (S1 Invitation Card). Results from the formative phase of this study [27] and evidence from the literature on notification slips [31] guided the development of the invitation card. The choice of the intervention to evaluate in the trial was guided by the formative phase of this study that was conducted in South Lunzu health centre on the assessment of strategies for MI in PMTCT. The study participants in the formative phase recommended an invitation card to the male partner as a more plausible strategy of involving males in PMTCT services. Furthermore, the participants suggested the wording of the invitation card [27]. Following enrollment and randomization, participants were either given an invitation card to extend to their partner or were asked to invite their partners through word of mouth.

### Participant recruitment: Randomization, Sequence generation, Concealment and Allocation

The recruitment period was from 14 June to 17 December 2013. Recruitment stopped when the last participant (number 462) was recruited in the study. Follow up activities completed on 24 February 2014. At the antenatal visit, women were informed about the study through a health education talk. Consecutively pregnant women were approached to solicit their willingness to participate in the study. Pregnant women who were interested and having consented into the study were sequentially randomized to the two groups using a permuted block design with randomly allocated block sizes of four, six and eight stratified by the study site with a 1:1 allocation ratio. Randomization list was computer-generated by the PI and the research Nurses recruited the participants. The allocation sequence was concealed from the enrolling nurse in sequentially numbered opaque sealed envelopes that contained individual randomization numbers with the specific group on a slip of paper. To enrol a pregnant woman in the study a research nurse would open the next consecutively numbered envelope and assign the treatment as per slip in it. The pregnant women in the intervention group were expected to pass on the invitation card that was sealed in an envelope and addressed to their partner while the pregnant women in the SoC group were expected to extend the invitation to their partner by word of mouth. Both groups were expected to return to the clinic after two and six weeks post enrolment. Women were encouraged to return to the clinic on the specified date irrespective of their male partner’s presence. At enrolment, a standardized questionnaire was administered to collect demographic information, medical and obstetrical history and HIV testing information.

### Study Follow up and Procedures

Study visits were conducted at week 2 and 6 from enrollment date. During these visits questionnaires were administered on whether the woman had invited her male partner, the reaction of the male partner after the invite, presence of a male partner at the clinic, the reason for non-reporting of a male partner to the clinic. Once a male partner presented himself for antenatal services with her partner, informed consent was sought from him as well as completion of socio demographic, past medical history, HIV testing details and ascertainment of future involvement with the other antenatal visits. No participant tracing activities were made in the study to avoid overestimation.

### Data Management

Data were double entered and managed using Microsoft access 2010. Data were screened to determine outliers, inconsistencies, strange patterns in the distribution in a spreadsheet and through simple descriptive analysis to determine completeness. Identified missing fields were checked against the files or other source documents within the clinic records. All records carried unique participant IDs and were stored in locked cabinets with limited access to study personnel and the electronic data was in a password protected computer.

### Ethical Approval

The protocol, consent forms and invitation card were approved on 3 June 2013 by the University of Malawi College of Medicine Research and Ethics Committee (COMREC) (identifier: COMREC No P 09/12/1279). Permission to conduct the trial in the two health centres was obtained from Blantyre District Health Office. All eligible women and their male partners provided a written informed consent (or a witnessed thumbprint if illiterate) prior to study participation. We recruited pregnant women and married from the age of 16 years because although they were under the age of the consenting age, which is 18, they are regarded as emancipated minors and are allowed to make a decision about their participation in a study. The married, pregnant women under the age of 18, provided their own consent without guardian or next of kin’s approval. The University of Malawi College of Medicine Research and Ethics Committee approved the protocol for the study population, and the consent procedures for the minors. The trial was registered with Pan African Clinical Trials Registry www.pactr.org (Identifier: PACTR No 201311000675100). We registered the trial with PACTR after participant enrolment began because we experienced financial problems with payment for the registration of the trial. The authors confirm that all ongoing and related trials for this intervention are registered.

### Statistical Analysis

The main strategy of analysis was the intention-to-treat analysis (ITT). In an intention-to-treat analysis, we compared all participants that were randomized in the intervention group with the SoC group according to the group that they were randomized. The pre-specified primary outcome was the proportion of pregnant women that were accompanied by their partners at week 2 and week 6 of the study after receipt of an invitation card from the health centre and the SoC group.

Descriptive statistics were computed for socio demographic variables to compare the participants in the two groups. The mean with standard deviation (SD) or median and interquartile ranges (IQR) were used for continuous variables where appropriate while categorical variables were summarized using proportions.

Unadjusted risk ratios (RR) with 95% confidence intervals (CIs) were computed to investigate the efficacy of the intervention. We did not adjust for any of the baseline characteristics because the two groups were comparable (Table 1). We conducted a random effects analysis to determine if there was any significant clustering by site of recruitment. The significance level of the statistical tests was set at 5%. The analyses were performed in Stata 12.0 software (Stata Corp, Texas, USA).

**Table 1:** Characteristics of Female participants (N = 462).

Characteristics	Intervention (n = 230)	Control (n = 232)	Total (N = 462)	P Values
**Age (years)**				
Median (IQR)	23 (20–28)	23 (20–27.5)	23 (20–28)	0.87
**Gestational Weeks**				
Mean (SD)	21.4 (5.2)	21.8 (5.0)	21.6 (5.1)	0.81
**Literate**				
Yes	183 (79.6)	177 (76.3)	360 (77.9)	0.39
No	47 (20.4)	55 (23.7)	102 (22.1)	
Employment Status*				
Not employed	200 (87.0)	197 (84.9)	397 (85.9)	0.77
Partner’s Employment Status*				
Formal	112 (48.7)	99 (42.7)	211 (45.7)	0.42
Partner’s Education Status*				
Secondary School Education	118 (51.3)	102 (44)	220 (47.6)	0.32
**Total Pregnancies**				
Primigravida	63 (27.4)	60 (25.9)	123 (26.6)	
Multigravida	137(59.6)	135 (58.2)	272 (58.9)	0.86
Grand multigravida	30(13.0)	37 (15.9)	67 (14.5)	
Household Characteristics*				
Latrine Available	224 (97.4)	227 (97.8)	452 (97.6)	0.75
Firewood cooking	147 (63.9)	161 (69.4)	308 (66.7)	0.21
No piped water	198 (86.1)	200 (86.2)	398 (86.2)	0.97
**Absence of Previous Pregnancy Complication**	213 (92.6)	211 (91.0)	424 (91.8	0.52
**HIV testing**				
Tested*	226 (98.3)	230 (99.1)	456 (98.7)	0.41
HIV infected	36 (15.9)	40 (17.4)	76 (16.7)	0.68
On ARVs	32 (88.9)	34 (85.0)	66 (86.8)	0.66

* = only the highest proportions have been presented, *therefore the figures are not adding up to N*

## Results

### Sociodemographic Characteristics

**Fig 1:**
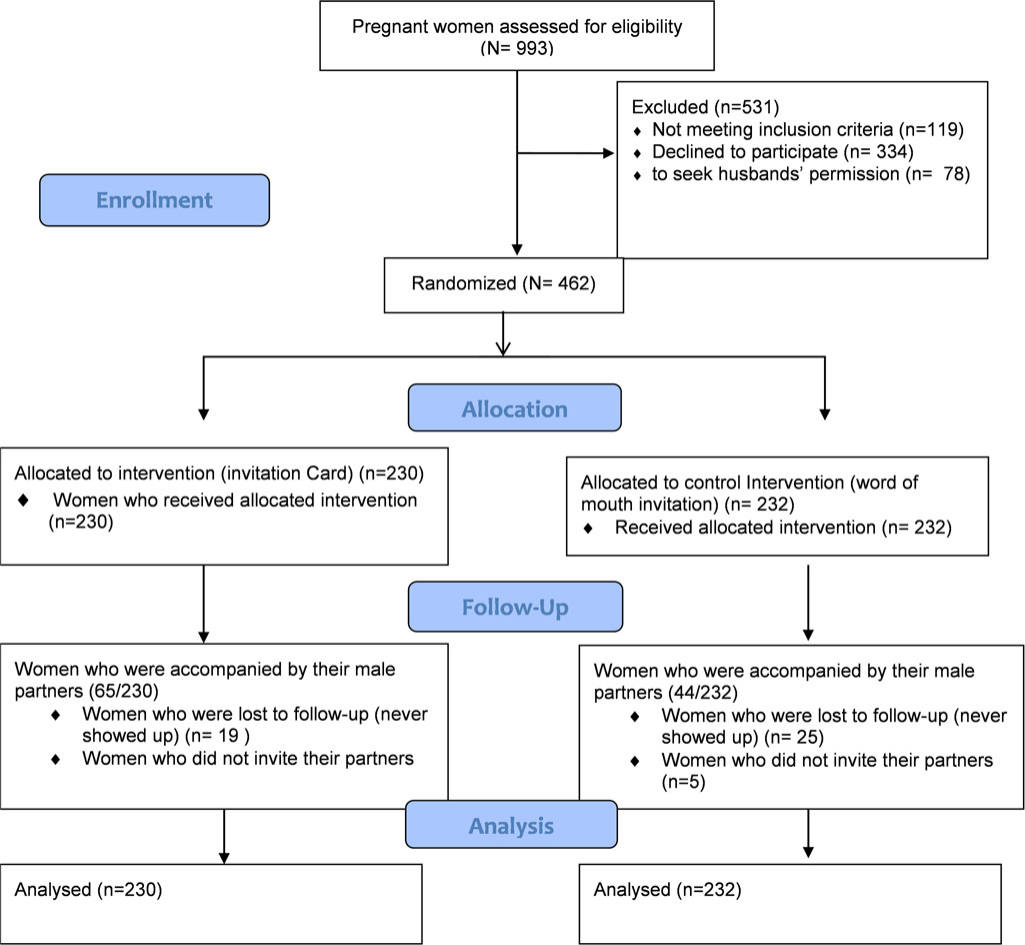
MI in PMTCT study flow according to Consort Flow Diagram showing the participants flow and numbers at each stage in the study from screening, enrollment, allocation, follow up and analysis from June 2013 to February 2014.

A total of 993 pregnant women were assessed for eligibility (Fig. 1) of whom 462 were eligible and randomized into the study and assigned to either intervention or SoC group with 230 randomized into the intervention group and 232 in the SoC group. Baseline socio demographic, obstetrical characteristics were comparable between the two groups (Table 1). The median age of the women in the intervention group was 23 (IQR: 20–28) years and 23 (IQR: 20–27.5) years for the SoC group. The mean gestation was 21.41 (SD: 5.15) weeks and 21.82 (SD: 5.00) weeks in the intervention and SoC groups respectively. The highest level of education for the women was a primary school education in both groups with 128 (55.7%) in the intervention group and 143 (61.6%) in the SoC group. In both the intervention and control groups, more participants were multigravidas with 137 (59.6%) and 135 (58.2%) respectively. On household items, most women in both groups reported to have a latrine 224 (97.4%) in the intervention group and 227 (97.8%) in the control group; most women had no piped water 198 (86.1%) in the intervention group and 200 (86.2%) in the control group and many used firewood for cooking 147 (63.9%) in the intervention group and 161 (69.4%) in the control group. Most women in the study arms, 213 (92.6%) in the intervention arm and 211 (91%) in the SoC arm had no history of previous pregnancy complication. More male partners’ to the women in both groups had a secondary school education as the highest level, with 118 (51.3%) in the intervention group and 102 (44%) in the SoC group. More male partners in both groups were formally employed with 112 (48.7%) in the intervention group and 99 (42.7%) in the SoC group. Overall, most women in the study, 458 (98.7%) had an HIV test and of these 76 (16.7%) were HIV infected, 36 (15.9%) in the intervention group and 40 (17.4%) in the control group. Of the HIV infected women 66 (86. 8%) were on ARVs, 3 (4.0%) were not yet on ARVs while 6 (9.2%) were loss to follow up and never reported for ART at the respective centres.

The socio demographic baseline characteristics of the women whose male partners reported to the antenatal clinic were comparable with those women whose male partners did not accompany them (Table 2) irrespective of randomization arm. Briefly, the median age for the women whose partners reported was 23 (IQR 17–29) years and 23 (IQR 20–27) years for the women whose partners did not report for PMTCT services. The mean gestation was 20.8 (SD: 5.4) weeks and 21.9 (SD: 5.0) weeks among the women whose partners reported and the group of women whose partners did not report respectively. The highest level of education for the women was a primary school education in both groups with 66 (60.6%) in the group of women whose partners reported and 205 (58.1%) in the group of women whose partners did not report for PMTCT services. In both groups, in the group of women whose partners reported and the group of women whose partners did not report for the PMTCT services, more women were multigravidas with 61 (56%) and 211 (59.8%) respectively. On household items, most women in both groups reported to have a latrine 106 (97.3%) in the group of women whose partners reported and 305 (97.7%) in the group of women whose partners did not report; most women had no piped water 93 (85.3%) in the group of women whose partners reported and 305 (86.4%) in the group of women whose partners did not report for the services and most also used firewood for cooking 81 (74.3%) in the group of women whose partners reported and 227 (64.3%) in the group of women whose partners did not report for PMTCT services. Most women 99 (90.8%) in the group of women whose partners reported and 325 (92,1%) in the group of women whose partners did not report for PMTCT services had no history of previous pregnancy complication. More male partners’ to the women in both groups had a secondary school education as the highest level, with 48 (44%) in the group of women whose partners reported and 172 (48.7%) in the group of women whose partners did not report for PMTCT services. More male partners in both groups were formally employed with 32 (38.5%) in the group of women whose partners reported and 169 (47.9%) in the group of women whose partners did not report for PMTCT services. All women who reported with their partners 109 (100%) had an HIV test while 347 (98.3%) of the women whose partners did not report for PMTCT services had an HIV test. Overall 76 (16.67%) were HIV infected, 21 (19.3%) in the group of women whose partners reported and 55 (15.9%) in the group of women whose partners did not report for PMTCT services. Of the HIV infected women 18 (94.7%) in the group of women whose partners reported were on ARVs compared to 39 (95.1%) of the women in the group whose partners did not report for PMTCT services.

**Table 2:** Characteristics of women whose partners reported versus women whose partners did not report to the Health Centres (N = 462).

Characteristics	Reported(n = 109)	Did not Report (n = 353)	Total (N = 462)	P Values
**Age (years)**				
Median (IQR)	23 (17–29)	23 (20–27)	23 (20–28)	0.05
**Gestational Weeks**				
Mean (SD)	20.8 (5.4)	21.9 (5.0)	21.6 (5.1)	1.0
**Literate**				
Yes	83 (76.2)	277 (78.5)	360 (77.9)	0.61
No	26 (23.9)	76 (21.5)	102 (22.1)	
Employment Status*				
Not employed	94 (86.2)	303 (85.8)	397 (85.9)	0.92
Partner’s Employment Status*				
Formal	32 (38.5)	169 (47.9)	211 (45.8)	0.20
Partner’s Education Status*				
**Secondary School Education**	48 (44.0)	172 (48.7)	220 (47.6)	0.45
**Total Pregnancies**				
Primigravida	28 (25.7)	95 (26.9)	123 26.6)	
Multigravida	61 (56.0))	211 (59.8)	272 (58.9)	
Grand multigravida	20 (18.4)	47 (13.3)	67 (14.5)	0.45
Household Characteristics*				
Latrine Available	106 (97.3)	305 (97.7)	451 (97.6)	0.77
Firewood cooking	81 (74.3)	227 (64.3)	308 (66.7)	0.05
No piped water	93 (85.3)	305 (86.4)	398 (86.2)	0.78
**Absence of Previous Pregnancy Complication**	**99 (90.8)**	**325 (92.1)**	**424 (91.8)**	**0.53**
**HIV testing**				
Tested*	109 (100.0)	347 (98.3)	456 (98.7)	0.17
HIV infected	21 (19.3)	55 (15.9)	76 (16.7)	0.40
On ARVs	18 (94.7)	39(95.1)	57 (95.0)	0.95

* = only the highest proportions have been presented, *therefore the figures are not adding up to N*

### Male Involvement

Of the 462 randomized women 109/462 (23.59%) came back with their partners at one visit. With respect to each study group, overall, 65/230 (28.26%) of the women in the intervention group reported with their partners while 44/232 (18.97%) women in the control group reported with their male partners In an intention to treat analysis, unadjusted analysis showed that women in the intervention group were significantly more likely to be accompanied by their male partners RR: 1.49 (95% CI, 1.06–2.09); p = 0.02) than women in the SoC group. We did not adjust for clustering by site of recruitment because our random effects model further suggested that there was no significant clustering by recruitment site with an inter cluster correlation coefficient (ICC) of 1.98x 10^-3^, (95% CI: 1.78 x10^-7^ - 0.96 x 10^-1^); p = 0.403 (Table 3).

**Table 3:** Unadjusted and Random Effects for the Primary outcome.

Type of Analysis	Unadjusted RR (95% CI)	P-Value	ICC	P-value
Intention to treat (n = 462)	1.49(1.06–2.09)	0.02	1.98 x10^-3^, (1.78 x10^-7^ - 0.96 x 10^-1)^	0.403
Per Protocol Analysis (n = 410)	1.43 (1.03–1.97)	0.03	7.87x10^-7^, (1.2 x10^-114^ - 1)	1

RR: Risk Ratio

CI: Confidence Interval

ICC: Inter cluster correlation coefficient

In a per protocol analysis, women in the intervention group were 43% more likely to be accompanied by their male partners than women in the SoC group, RR: 1.43 (95% CI, 1.03–1.97), p = 0. 03. We did not adjust for clustering by site of recruitment because our random effects suggested no evidence of clustering by recruitment site with ICC of 7.87 x10^-7^, 95% CI: 1.2 x10^-114^ - 1); p = 1.00 (Table 3).

## Discussion

The main finding from this randomized controlled open label trial on the use of an invitation card as a male involvement strategy in PMTCT services was that an invitation card significantly increased the proportion of women who were accompanied by their male partners for the PMTCT services when compared to the women who used a word of mouth invite.

The efficacy of the invitation card in this study was consistent with a randomized trial done in Uganda by Byamugisha et al [37] who demonstrated that significantly more men reported to the antenatal clinic following administration of an invitation letter or information letter which had details of the antenatal care services provided including relevance and costs of PMTCT and couple counseling services or information letter which had general information of antenatal care services without personalization. The study in Uganda however, did not show any significant differences between the proportions of men who accompanied their partners between the two groups. Similarly, in a randomized trial in South Africa by Mohlala et al. [25] reported that women in the group that extended written voluntary counseling and testing invitations to their male partners were 36% more likely to be accompanied by their partners than the women in the group that extended patient information sheets to their partners. Likewise, our study results mirror results observed in the management of STIs in Malawi where provider assisted partner notification led to more partners reporting to the clinic for treatment and eventually improved referral to other health services [31]. Our results coupled with the previous studies suggest that an invitation card is a feasible means of incorporating men in PMTCT services.

The increased uptake of the invitation card by men in this study may partially have resulted from implementing a strategy that men proposed as an effective method of inviting them in the formative study prior to the trial [27]. In the formative study men preferred an invitation card that does not specify about HIV testing addressed to them because it personalizes the message [27]. On the other hand the fear of learning one’s HIV status, socioeconomic obligations as earlier suggested in the formative phase of this study [38], and also by Morfaw et al [39] in their systematic review on barriers to MI in PMTCT may explain the non-uptake of the intervention by the male partners who did not accompany their spouses.

The higher proportion observed in the uptake of HIV testing and ARVs for PMTCT point to the effectiveness of the PMTCT services in the health centres which is partially due to the health system in Malawi which rarely permits a woman to pass through an antenatal clinic without an HIV test [40]. However, the results also highlight the missed opportunities as evidenced by the proportion of women who navigated through the system without an HIV test. The HIV testing and HIV infection proportions in this study are higher than proportions reported in the Malawi Quarterly report (MQR) for January-March 2014 where 82% of antenatal attendees were HIV tested with 7.8% HIV infected[41]. The proportion of ART uptake in this study was lower than the 93% reported in the January-March 2014 MQR [41]. The lack of HIV testing and non- initiation of ART could be partially explained by the lack of male support in the service [14]. The loss to follow up of HIV infected women who had not initiated ARVs highlights the challenges with initiation and retention in a PMTCT programme. This finding remains consistent with findings from review of studies that highlighted that participants are lost in pre ART [42] at different cascades of PMTCT[3,43].

The strength of this study is that it was a randomized controlled trial, thereby controlled for known and unknown factors that could have confounded the results.

### Limitations

As the intervention was implemented on married, pregnant women with a gestation age equal or less than 30 weeks and in two health centres in Blantyre, the results may only be applicable to women with such specifications. Our results are based on the assumptions that all women in the study unless specified invited their partners. Although participants were from the designated catchment areas of the Health Centres, we did not collect data on the distance between the participants home and the health centre. Further research may look at impact of distance of MI in PMTCT.

### Implications

The results of this study imply that a simple intervention like an invitation card passed through a pregnant woman to her partner may increase men’s participation in PMTCT services. We recommend the adaptation of an invitation card to male partners into the PMTCT policy as a strategy for increasing MI in PMTCT services.

## Supporting Information

S1 CONSORT Checklist (DOC)

S1 Informed Consent Form Informed Consent Form for Females. (PDF)

S2 Informed Consent Form Informed Consent Form for Males. (PDF)

S1 Invitation Card (PDF)

S1 Protocol Trial Protocol. (PDF)
