# Supplementary material for: Invitation Cards during Pregnancy Enhance Male Partner Involvement in Prevention of Mother to Child Transmission (PMTCT) of Human Immunodeficiency Virus (HIV) in Blantyre, Malawi: A Randomized Controlled Open Label Trial
Source: PLoS One. 2015 Mar 3;10(3):e0119273. doi: 10.1371/journal.pone.0119273 (PMC4348422; doi:10.1371/journal.pone.0119273)
Supplement: S2 Informed Consent Form — (PDF) [file pone.0119273.s003.pdf]

Informed Consent Form For Males

College Of Medicine, Department of Community Health

Informed Consent Document- Male Partner Enrollment Consent Form

---

Study Title: Feasibility of Male Involvement in Prevention of Mother to Child Transmission of HIV Services in Blantyre, Malawi.

Principal Investigator: Alinane L.M. Nyondo, RN/M,BN-AP, MN-CH

PI Version Date: 14 May 2013

What you should know about this study

You are being asked to join a research study because you have reported to the clinic with your partner following an invite. This consent form explains the research study and your part in the study. Please read it carefully and take as much time as you need. Please note that your participation in this study is entirely voluntary and you may decide not to take part or to withdraw from this study at any time. There will be no penalty if you decide to quit the study. During the study, we will tell you if we learn any new information that might affect whether you wish to continue to be in the study or not.

This study is being done in fulfillment of a doctorate degree in Public Health at the College of Medicine in Blantyre, Malawi. The Investigator of this study is Alinane Nyondo. Before you decide if you want to be a part of this study, we want you to understand the study.

The study staff will talk with you about this information. You are free to ask questions about this study at any time. Before you decide whether to take part in this research study, you need to know the purpose, the possible risks and benefits to you, and what will be expected of you during the study. After the study has been fully explained to you, you can decide whether or not you want to participate. Once you understand this study, and if you agree to take part, you will be asked to sign this consent form or make your mark in front of someone. You will be offered a copy of this form to keep.

Purpose of research Study

The purpose of this study is to see if using invitation card as a strategy for MI in PMTCT increases the uptake of the PMTCT services by men and women. Women will be randomly allocated to a group that uses an invitiation card or a group that does not use an invitation card . This study will compare the proportion of men accompanying their partners/wives for PMTCT services in the group that uses an invitation card with the proportion of men that accompany their wives/partners in the group that is not using the invitation card. This study will also asses the rates of uptake of PMTCT services and PMTCT specific education where necessary in women in both study groups.

Procedures

If you agree to join this study, you will be asked to come back to the study clinic at study week 6 following your partners/wife’s schedule. During the initial study visit we will ask you to answer a few questions about yourself such as your age, tribe, religion, where you stay, if you have tap water and electricity at home, your occupation , health history, uptake of PMTCT services and any other relevant information on your health available in the health records. About 462 pregnant women will take part in the study.

Risks and or discomforts

We do not anticipate major risk. Some questions may be sensitive however we would like you to answer them because it will help us to understand the best way to handle MI in PMTCT. Your visits here will take place in private. We will make every effort to protect your privacy and confidentiality while you are in the study and will not use your name in the study summaries.

Potential Benefits

There may be no direct benefit to you from this study, but the information we collect may help the health workers improve the way it delivers health care to women and their partners in PMTCT services in the future.

Reasons why you may be withdrawn from the study without your consent

You may be removed from the study without your consent for the following reasons:

- The study is stopped or cancelled by the Malawi Ministry of Health, the Malawi College of Medicine Research Ethics Committee.
- Staying in the study would be harmful to you.
- You are not able to attend study visits or complete the study procedures
- Your partner has decided to quit the study

Costs and Compensation

There will be no cost to you for study-related visits. At the end of each visit, you will receive reimbursement for transport to and from the clinic. If you choose not to take part in this study, it will have no effect on your regular health care at this hospital.

Confidentiality

Efforts will be made to keep your study records and test results confidential to the extent permitted by law. However, we cannot guarantee absolute confidentiality. You will be identified by a code, and personal information from your records will not be released without your written permission except to health care providers when needed. Any publication of this study will not use your name or identify you. The study records will be kept in a locked cabinet at the clinic and later at the College of Medicine. However, your records may be reviewed by study staff, study monitors, the Malawi Ministry of Health, and the Malawi College of Medicine Research Ethics Committee (COMREC).

All study documents will be stored in locked cabinets in the study clinic and at the College of Medicine . The database will be maintained in a password protected computer with access limited to the study team only. All the consent forms and study records will be destroyed after all academic purposes have been met.

Research Related Injury

It is unlikely that you will be injured as a result of being in this study, however if you are injured as a result of being in this study, the study staff will give you immediate necessary treatment for your injuries. You will not have to pay for this treatment. You will then be told where you can get additional treatment for your injuries, if needed. There is no program for monetary compensation or other forms of compensation for such injuries through this study. You do not give up any legal rights by signing this consent form.

Problems or Questions

For questions about this study or a research-related injury, contact:

- Ms Alinane Linda Nyondo, Principal Investigator
- Located at the College of Medicine, Department of Community Health, phone 0888 202 670

For questions about your rights as a research subject, contact:

- The Secretariat, College of Medicine Research Ethics Committee
- Located at the Malawi College of Medicine, phone 01-874-377

SIGNATURE PAGE:

MI in PMTCT STUDY Version 3.0 dated 14 May 2013

If you have read the informed consent, or have had it read and explained to you, and understand the information, and you voluntarily agree to join the study, please sign your name or make your mark below.

Participant Name (print)	Participant Signature **or thumbprint**	Date
Study Staff Conducting Consent Discussion (print)	Study Staff Signature	Date
Witness Name (print) (As appropriate)	Witness Signature	Date
