# Supplementary material for: Invitation Cards during Pregnancy Enhance Male Partner Involvement in Prevention of Mother to Child Transmission (PMTCT) of Human Immunodeficiency Virus (HIV) in Blantyre, Malawi: A Randomized Controlled Open Label Trial
Source: PLoS One. 2015 Mar 3;10(3):e0119273. doi: 10.1371/journal.pone.0119273 (PMC4348422; doi:10.1371/journal.pone.0119273)
Supplement: S1 Invitation Card — (PDF) [file pone.0119273.s004.pdf]

Appendix 1- Invitation Card

College of Medicine - Blantyre DHO
South Lunzu and Mpemba Health Centres

To: (Partners' PID) ____________________

Your wife is accessing antenatal care at ________________________Health Centre. We kindly ask you to accompany her at her next antenatal visit on________________ so that we counsel both of you on her care while pregnant.

If you have any questions please call Ms Alinane Nyondo on this number 0888-202-670/ 0999 44 1212 or ask a Nurse at ___________________ Health Centre
