# Supplementary material for: Invitation Cards during Pregnancy Enhance Male Partner Involvement in Prevention of Mother to Child Transmission (PMTCT) of Human Immunodeficiency Virus (HIV) in Blantyre, Malawi: A Randomized Controlled Open Label Trial
Source: PLoS One. 2015 Mar 3;10(3):e0119273. doi: 10.1371/journal.pone.0119273 (PMC4348422; doi:10.1371/journal.pone.0119273)
Supplement: S1 Protocol — (PDF) [file pone.0119273.s005.pdf]

UNIVERSITY OF MALAWI, COLLEGE OF MEDICINE, COMMUNITY HEALTH DEPARTMENT

THE FEASIBILITY OF MALE INVOLVEMENT IN THE PREVENTION OF MOTHER TO CHILD TRANSMISSION OF HIV SERVICES IN BLANTYRE MALAWI

Alinane Linda Nyondo Mipando, RNM, BN-AP, MN-CH

Supervisors:

Associate Professor Adamson Muula- College of Medicine

Associate Professor Angela Chimwaza- Kamuzu College of Nursing

Table of Contents

EXECUTIVE SUMMARY **4**

CHAPTER 1 INTRODUCTION **6**

PREVENTION OF MOTHER TO CHILD TRANSMISSION OF HIV SERVICES UPTAKE IN MALAWI 7
1.1 PROBLEM STATEMENT 7
1.2 SIGNIFICANCE OF THE STUDY 8
STUDY OBJECTIVES 8

CHAPTER TWO LITERATURE REVIEW **10**

2.1 INTRODUCTION 10
2.2 DEFINITION OF MALE INVOLVEMENT 10
2.3 PREVENTION OF MOTHER TO CHILD TRANSMISSION OF HIV AND MALE INVOLVEMENT 11
2.4 BARRIERS TO MALE INVOLVEMENT 12
2.5 BENEFITS OF MALE INVOLVEMENT 14
2.6 STRATEGIES FOR MALE INVOLVEMENT 16
2.7 LITERATURE REVIEW OF STUDIES DONE IN MALAWI 18
2.8 CONCEPTUAL FRAMEWORK- PRECEDE-PROCEED MODEL OF HEALTH PROGRAM PLANNING AND EVALUATION 22
2.9 CONCLUSION OF LITERATURE REVIEW 24

CHAPTER 3 METHODS: INTERVENTION PHASE **25**

3.1 INTRODUCTION 25
3.2 STUDY DESIGN: RANDOMISED CONTROL TRIAL 25
3.3 RECRUITMENT AND ENROLLMENT PROCEDURES 26
3.5 STUDY POPULATION 28
3.6 SAMPLE SIZE AND ACCRUAL 29
3.7 SELECTION OF PARTICIPANTS: INCLUSION AND EXCLUSION CRITERIA 29
3.8 DATA COLLECTION 30
3.9 DATA MANAGEMENT 31
3.10 DATA ANALYSIS 32
3.11 STUDY PERIOD 33
3.12 RESULTS PRESENTATION 34
3.13 DISSEMINATION OF RESULTS 34
3.14 ETHICAL CONSIDERATIONS 34
3.15 POSSIBLE CONSTRAINTS 36
3.16 STUDY REQUIREMENTS 36
3.17 BUDGETARY ESTIMATES 38
3.18 JUSTIFICATION OF THE BUDGET 39

BIBLIOGRAPHY **41**

List of Tables

Table 1- Evaluations during study visits 30

List of Figures

Figure 1 - The Authors' application of the PRECEDE PROCEED Model to the study 23
Figure 2- Randomization into the two arms in each Health Centre 27

Executive Summary

Type of Research

This is a randomized open label trial among pregnant women in the antenatal clinics to determine the feasibility and effectiveness of an invitation card as a strategy for Male Involvement (MI) in Prevention of Mother to Child Transmission (PMTCT) of Human Immunodeficiency Virus (HIV) services on the uptake of the services. Randomization will be stratified by health centre. Pregnant women will be enrolled at any time point in pregnancy, until 30 weeks gestation. The study will have two arms, Arm A and Arm B. Arm A will be the intervention arm where women will be randomized to use of an invitation card as a strategy for MI in PMTCT of HIV while in Arm B, the non-intervention arm, women will not use the invitation card. Data will be collected through structured questionnaires from eligible, consented, and randomized participants over several visits.

Problem

Mother to child transmission (MTCT) of HIV accounts for 14% of all new HIV infections in the world and it also accounts for most of the infections in children in Sub Saharan Africa. The Malawi Ministry of Health estimates that about 72 450 HIV- exposed infants are born in Malawi every year. Despite the scaling up of PMTCT services, they are not fully utilized by the women. One of the reasons for the lack of full utilization of the services is the lack of male involvement in the services. Incorporating male partners in PMTCT is important because it has been shown to improve the uptake and promote a positive attitude towards the service. Several studies have cited lack of MI as a reason for women dropping out of a PMTCT program. While there are considerable suggestions on the strategies, limited data exist to show feasibility of MI in PMTCT services. The current PMTCT guidelines for Malawi have not explicitly spelt out the strategy for MI in the service. As Malawi is aiming at the elimination of paediatric HIV infection, removing all barriers to such a goal becomes paramount and MI may be another strategy towards that goal.

Broad Objectives

To evaluate the effectiveness and feasibility of an Invitation card to male partners for MI in PMTCT services.

Primary Objective

1. To compare proportions of pregnant women that are accompanied by their partners at week 2 and week 6 of the study after receipt of an Invitation card from the Health Centre and the non-intervention study arm.

Secondary Objectives

1. To compare the proportion of male partners taking up HIV testing in both study arms at study week 2 and 6.
2. To **compare proportion** of women taking up PMTCT services such as HIV testing, Antiretrovirals (ARVs) for PMTCT, Infant feeding Options in both arms at **study week 2 and 6**.
3. To **compare proportion** of women adhering to treatment by using pill count and keeping appointment dates in both study arms at **study week 2 and 6.**

Methodology

Male Involvement in PMTCT is a multiple sequential design study with two phases. The first phase was a qualitative part with focus group designs and Key informant interviews. Phase 2 is a randomised open label facility based trial among pregnant women in the antenatal clinics to determine the effectiveness and feasibility of an invitation card to male partners as a strategy for MI MTCT of HIV services on the uptake the Services. Pregnant women will be enrolled at any time point in pregnancy but before 30 weeks gestation. The study will have two arms, Arm A and Arm B. Arm A will be the intervention arm where women will be randomized to the MI strategy “an invitation card” while in Arm B, the non-intervention arm women will not use the strategy. Approximately 462 pregnant women will be enrolled with 231 into each arm. They will be followed up for 6 weeks and will have 2 follow up visits.

Expected Findings and Dissemination

In the proposed study we aim to determine the feasibility and effectiveness of using an invitation card as a strategy for MI in PMTCT on the uptake of PMTCT services by both women and men. The information obtained will be summarised in manuscripts and will be submitted to peer reviewed journals. In addition, Policy Briefs and dissemination of findings will be held with the Ministry of Health, Blantyre City Assembly and Blantyre District Health Office. The results will also be shared in academic sessions such as journal clubs and/or research in progress sessions within the College of Medicine and its affiliates. The results will be used as needed to inform local policy on MI in PMTCT which will eventually assist in the incorporation of males in the service. The results will also be disseminated through conferences locally or internationally.

CHAPTER 1 INTRODUCTION

Human Immunodeficiency Virus and Acquired Immunodeficiency Syndrome (HIV and AIDS) remain a burden in Sub Saharan Africa which has 68 % of the entire global HIV and AIDS burden. (1). Human Immunodeficiency Virus and Acquired Immunodeficiency Syndrome continues to heavily affect women and children worldwide. It has been reported that more than half of all HIV infections are in women and girls(1). It is further estimated that 76% of all infected women in the world live in Sub Saharan Africa where women between 15-24 years are eight times more likely to be HIV infected than their male counterparts (1). Most of the infections in adults primarily occur secondary to unprotected heterosexual relationships (1).

Mother to child transmission (MTCT) of HIV accounts for 14% of all new HIV infections in the world and it also accounts for most of the infections in children in Sub Saharan Africa (2). World Health Organization (WHO) advocates for a comprehensive approach in the prevention of MTCT. Currently, WHO recommends that HIV infected pregnant women and their infants receive Antiretrovirals (ARVs) as a measure for PMTCT (3)

Implementation of ARVs for PMTCT has made progress both in the ARV regimen provided as well as uptake of the intervention by the women. In 2009, it was estimated that in Sub Saharan Africa, 54% of HIV infected pregnant women received a combined regimen of antiretrovirals and 30% received single dose Nevirapine for PMTCT of HIV. In other countries in Africa, such as Botswana, Namibia, South Africa and Swaziland, antiretroviral coverage for PMTCT was more than 80% while in West and Central Africa coverage has been as low as 23% in 2009 (1).

In Malawi, the HIV prevalence is at 11% in adults within 15-49 age groups while among women of the same age group it is at 13% versus 8 % in men. From the earlier Demographic Health surveys conducted in 2004 and 2010 by the National Statistics Office (NSO), prevalence in women has remained static at 13% from 2004 to 2010 (4). Regionally, HIV prevalence among women is high in the Southern region of Malawi where it is 17.6% (4). Similarly to the world's statistics, women and adolescent girls are four times more likely to be infected than men and that women tend to be infected whilst young when compared with men (5). The high prevalence in these groups is attributed to the weak status of women and girls because of economic, cultural, and social factors that predispose young women to HIV and AIDS at a younger age(6).

Malawi launched PMTCT of HIV services in 2003 with an aim of curbing peadiatric HIV Infection in order to have an HIV free generation and also to promote the health status of HIV exposed infants and their families

(7). The Malawi Ministry of Health further estimates that about 72 450 HIV- exposed infants are born in Malawi every year (7). Additionally National AIDS Commission (NAC) in the United Nations General Assembly Special Session (UNGASS) 2008-2009 report, projected that there will be about 97, 872 women who will require PMTCT interventions thereby increasing the number of HIV exposed infants born in the country (5). Malawi has about 542 facilities offering ANC services (8). Prevention of Mother to Child Transmission of HIV services in Malawi are offered within the Maternal and Child Health Service and are mostly utilized by women. Malawi uses an Opt Out approach (Provider initiated routine antenatal testing with group education in HIV and PMTCT) in HIV testing for all women presenting at the ANC clinics in which the patient must actively refuse the test. The services offered include HIV Pretest and Post Test counseling, HIV testing and Infant feeding counseling. The services also include administration of Anti retrovirals to the woman while pregnant, in labour and postnatally and also to the infant and referral of infant for ARVs if HIV infected.

Prevention of Mother to Child Transmission of HIV Services uptake in Malawi

The uptake of PMTCT services in Malawi has progressed steadily. The United Nations joint programme on HIV/AIDS (UNAIDS) estimated that in 2009, 52% of the pregnant women in Malawi had an HIV test (9) while in 2010, the estimate was at 66% and that between 15-23% received effective ARVs for PMTCT. The estimates further indicated that in 2010, Malawi achieved 6% towards the goal of providing ARVs for PMTCT to 90% of HIV infected pregnant women (10). The prevalence of HIV among pregnant women is estimated at 12%. In 2006, WHO estimated that coverage of PMTCT services was 27% while HIV testing in pregnant women in 2009 was at 52% (9). In 2009, UNICEF estimated that 58% of HIV infected pregnant women used PMTCT services in Malawi (11). Reports from the Ministry of Health in Malawi indicate that between 2010-2012 on average 94% of the women presented with a valid HIV result in labour which was suggestive of earlier antenatal testing (8, 12, 13). Among HIV infected pregnant women attending the maternity services from 2010-2012, on average 83% of the women received ARVs during labour respectively. The coverage for ARV prophylaxis has been static at 43% since 2011 to date (13). Ninety- two percent of HIV exposed infants received ARV prophylaxis in 2010 - 2011 which translated into 32 % - 43%, coverage within the estimated population of infected children , and has been on 43% since 2011 to date (8, 12, 13).

1.1 Problem Statement

Despite the scaling up of PMTCT services, they are not fully utilized by the women. One of the reasons for the lack of full utilization of the services is the lack of male involvement in the services. Incorporating male

partners in PMTCT is important because it has shown to improve the uptake and promote positive attitude towards the service. Several studies have cited lack of MI as a reason for women dropping out of a PMTCT program (14-16). The current way of measuring male involvement has been criticised as narrow and that it needs further development from men accompanying women for their appointments to include aspects of the natural social support by male partners since not all male partners would have the woman's health as a priority (17). In as much as there has been evidence on the need of MI from literature, active strategies have not been put in place to include males in the services. While there are considerable suggestions on the strategies, limited data exist to show feasibility and effectiveness of MI in PMTCT services. Although the current PMTCT guidelines for Malawi are in support of MI in the service, the strategy for MI has not been explicitly spelt out. As Malawi is aiming at the elimination of paediatric HIV infection, elimination of all barriers to such a goal becomes paramount and MI may be another strategy towards that goal.

The purpose of this study is to evaluate the effectiveness and feasibility of an Invitation card to male partners for MI in PMTCT services in Blantyre, Malawi.

1.2 Significance of the Study

It is hoped that the proposed research, test a strategy that can be used to incorporate males in PMTCT services. This strategy may improve involvement of men in PMTCT which is believed to eventually improve the uptake of PMTCT services by women. An increase in the uptake of PMTCT services will eventually lead to a reduction in peadiatric HIV and also improvement in maternal health. The results from the study may inform policy on how to operationalise MI in PMTCT services which may potentially increase the uptake of the service by the women.

STUDY OBJECTIVES

1.3.3.1 Broad Objective

To evaluate the effectiveness and feasibility of an invitation card as a strategy for MI PMTCT services.

1.3.3.2 Primary Objective

1. To compare proportions of pregnant women that are accompanied by their partners at week 2 and week 6 of the study following receipt of an invitation card in the intervention and the non-intervention study arm.

1.3.3.3 Secondary Objectives

1. To compare the proportion of male partners taking up HIV testing in both study arms at study week 2 and 6.
2. To compare proportions of women taking up PMTCT services such as HIV testing, ARVs for PMTCT, Infant feeding Options in both arms at study week 2 and 6**.**
3. To compare proportions of women adhering to treatment by using pill count and keeping appointment dates in both study arms at study week 2 and 6.

CHAPTER TWO LITERATURE REVIEW

2.1 Introduction

This section outlines selected relevant literature on MI in PMTCT. It starts with literature from around the world and ends with Malawi specific literature. It is divided into sub sections under Definition of MI, PMTCT and MI, barriers to MI in PMTCT, benefits and strategies for MI. It also presents the Conceptual framework that guides the study. The aim of the literature review is to highlight and discuss what is already known on MI in PMTCT and also to expose the gaps in knowledge on MI in PMTCT.

2.2 Definition of Male Involvement

Definition of MI varies with varying disciplines and programmes. United Nations Fund for Population Activities (UNFPA) in 1995 defined MI as support from men to women on what women are involved in (18). Male Involvement also takes the form of encouraging a partner to take an intervention. Nonetheless, Helzener (1996) argued that there is another form of involvement that renders a woman powerless in aspects of decision making (19). In the context of PMTCT, MI includes men's attendance of antenatal care clinic and also undertaking an HIV test within the ANC (20). In a South African study women defined involvement as support they received from male counterparts, this support included provision of a) resources such as transport money or means of access for a woman to attend antenatal services, provision of food while at the clinic and provision of infant supplemental or replacement feeds b) Reminding the woman of the PMTCT appointments, compliance with ART medication and also encouraging on testing the infant for HIV, c) emotional support after learning of HIV positive status, d) decision making on the mode of infant feeding option to follow (21), e) assisting the woman at home with household chores, f) interacting with the ANC providers when discussing his partner's health care issues (22).

In this study Male involvement in PMTCT implies the following aspects

- A man accompanying his partner for ANC services where PMTCT is discussed.
- A man attending to the PMTCT sessions with his partner.
- A man offering emotional support to his partner.
- A man and his partner counseled for HIV testing together and taking the test together.
- If unwilling to take a test a man should at least allow his partner to take a test without negative consequences.
- A man supporting and encouraging his partner to follow through with the PMTCT interventions.

2.3 Prevention of Mother to Child Transmission of HIV and Male Involvement

Prevention of Mother to child transmission of HIV (PMTCT) is the major way of reducing the rate of Paediatric HIV Infection. Mother to child transmission rate in the absence of any antiretroviral intervention is at 15-45 %, 8-10% with short course antiretroviral intervention and it is less than 5% with triple antiretroviral regimen (1, 3, 23). Despite the documented importance and increased benefits of PMTCT services, WHO in 2007 estimated that the uptake in Sub Saharan Africa was as low as 11%, it ranged from 8-54% showing the difference in levels of uptake of the service in the region (24, 25). United Nations Programme on HIV/AIDS further reported that, in 2007, only 33% of HIV Infected women received the required treatment and in low and middle income countries only 21% of pregnant women had an HIV test as part of their antenatal care (1). One of the reasons cited by several studies for the low uptake of PMTCT services, such as the low numbers of pregnant women undertaking an HIV test and also the low uptake of interventions such as ART for PMTCT, is the lack of or the low levels of male involvement (MI) in the services (14, 15, 26).

Male Involvement in PMTCT is considered crucial in a family setting where men are the decision makers as is the case in most African countries (26). Studies have stated that a household head, who in most cases is the husband, greatly influences the woman's ability in seeking health care or implementing health practices and interventions (27-29). Women in Tanzania reported that it would be easier for men to ask or take the women to the clinic for couple counselling on HIV because men are decision makers in most homes (30). Additionally, studies in several African countries argue that male partners have a role in the woman's risk of acquiring HIV and also in the uptake of HIV testing and MTCT prevention programmes (31-34). A study in Kenya suggested that involvement of partners in HIV testing is necessary for increasing uptake of interventions for reducing perinatal HIV transmission (35). It has been further asserted that the success of prevention of vertical transmission depends on cooperation within a couple because a woman's partner has a strong influence on the woman's uptake of VCT (31). Thus, it was not surprising to note that health workers in a Tanzanian study suggested the inclusion of male partners in PMTCT services in order to increase and improve PMTCT uptake and follow up (36). Consequently, it is not surprising that lack of MI was cited as a reason by some women in Botswana for withdrawing from a PMTCT program, and also that some women did not enrol into the program because they feared negative consequences such as domestic violence (37).

Husband's involvement in form of his approval to a service is directly related to a woman's uptake of an HIV test (38, 39). An association was observed between male partner involvement in antenatal HIV testing and increased uptake of interventions for prevention of vertical and sexual transmission of HIV. Women, whose partners were involved, received Nevirapine during their follow up visits, avoided breastfeeding their babies, adhered to the infant feeding method chosen and reported condom use more than those whose partners were not involved (34, 38). Consequently, it was noted that couple counselling improved the uptake and adherence to the PMTCT programme although its success is affected by the low numbers of men who attend antenatal care with their partners (38). Similarly, a review of studies on the effect of paternal involvement on utilisation of perinatal care services showed that paternal involvement resulted into utilisation of prenatal services such as early receipt of prenatal care (40). This concluded that strategies for male involvement were urgently required for an increased uptake of PMTCT services (34).

2.4 Barriers to Male Involvement

Studies done in Africa have however shown that efforts to involve men in ANC services have only resulted in a few husbands being involved (38, 41, 42). PMTCT services have been criticized of only focusing on females hence sidelining males who are the primary support unit to the woman (43). Literature has categorised the barriers to MI into: Health systems, Gender/Cultural, Health workers attitudes, lack of knowledge, Stigma and violence and socioeconomic factors.

2.4.1 Health Systems

Hospital policies are a barrier to MI in PMTCT Services. In Nepal, secondary to a hospital policy, men were not allowed into the labour and delivery area, this hindered their involvement in the maternal services (44). The antenatal clinic is regarded as a women's area in most areas, and this impact negatively on MI. In DRC, men were unable to use the Antenatal clinics or to participate in PMTCT programs because they are located in women's domain (45) while in Tanzania men were not involved in the PMTCT services because they were not comfortable with the services being offered within women's domain (46).

The method of delivering PMTCT services has created a barrier for MI in the service. For instances the use of women to relay the health information to men or inviting a male partner to the clinic through a message passed through the wife has resulted in lack of involvement by men because this method undermines some

cultural pillars on gender roles, where a woman does not instruct a man on what to do; and also the non-inclusion of men from the outset of PMTCT programmes has created a gap in MI in the programme (47). The distance to the clinic deterred men from being involved in antenatal care of their partners, most men would not accompany their partners if the distance to a health facility was long (48). User fees also hinder men from being involved in PMTCT services. In Uganda, men reported that in instances where the PMTCT services attract a fee, men would refrain from getting involved, in a way avoiding the financial responsibility (49).

2.4.2 Health Worker Attitudes

Literature has shown that the attitude of the health workers limited male involvement in health services such as in antenatal care. A study conducted in Uganda showed that men were not involved in the health care of their wives because of the impolite nature of health workers (49).

2.4.3 Cultural Factors

Culturally, it is strongly believed that pregnancy and raising a child is a woman's responsibility automatically hindering MI in pregnancy issues because men are not expected to take part. In some instances men failed to accompany their wives for antenatal services because culturally it was incorrect to follow one's partner for antenatal care (34, 49). A study done in Uganda showed that, men were willing to take an HIV test however their willingness was not followed by a corresponding action because most men considered accompanying a wife to the antenatal clinic as a strange behavior (50). Polygamy is a barrier to MI in PMTCT services. In polygamous cultures, such as in Cameroon, it was not feasible for a husband to attend PMTCT services with each wife. It was feared that if he accompanied one and not the others; it would fuel up disagreements between the wives (51, 52).

2.4.4 Unwillingness and Fear of knowing one's Status

Lack of MI has been attributed to the unwillingness by men in learning their HIV status indirectly; the understanding is that once the partners' status is known then men can use their partners' status as a proxy (35). Secondary to this fear, a study in Zimbabwe noted that men disassociated themselves from any services related with HIV and AIDS which resulted in them hindering their partners in accessing the services as well (53).

2.4.5 Limited Knowledge on PMTCT /Pregnancy Issues

Inadequate knowledge by men on their expected responsibility and role in maternal child health services is a prominent barrier to MI in the services and this barrier is aggravated in instances where men consider the services be for females only (44, 48, 54, 55). In addition to the cultural inappropriateness of men being involved in the pregnancy care of their partners, studies have also reported that some men felt they cannot be involved because they do not possess the required skill to support their spouses during pregnancy (56, 57).

2.4.6 Stigma and violence

A review of studies reported a lack of disclosure of HIV status by pregnant women for fear of violence, stigma thus affecting male involvement in the service because men were not aware of the need of their involvement (58). Additionally, men were not involved in the maternal health services of their partners because of the societal stigma that is attached to that. In Nepal, men especially the older generation were not involved because the society does not respect nor does it encourage men to be involved during pregnancy. Such men in this society are regarded with derogatory terms implying one who takes orders from a wife or someone who has been “hen pecked” (44).

2.4.7 Socio economic factors

The need to work and provide for the family hindered MI in PMTCT services especially when services are offered during working hours only. Some men expressed time constraints to attend to the PMTCT service (43, 49).

2.5 Benefits of Male Involvement

Literature describes several benefits for MI in PMTCT on the different cascades of PMTCT. The benefits are discussed in relation to uptake of a) antenatal care, b) PMTCT prophylaxis, c) HIV testing, and d) health information sharing within partners. It was reported that men who were aware of PMTCT programmes were twice as likely to be involved in PMTCT service (49).

2.5.1. Antenatal Care Uptake

Male Involvement resulted in an increase in antenatal care uptake at different levels and aspects of antenatal care. Women who attended antenatal care with their partners were more likely to initiate antenatal care in the first trimester compared to those whose partners were not involved. The involvement of a spouse also had a positive impact on the health of the partner, pregnancy and child. Thus, MI has a positive impact on a partner's health such as reduction of smoking in pregnancy (59-63).

2.5.2 Uptake and Adherence to Prophylaxis for Prevention of Mother to Child Transmission of HIV

Male involvement increases the uptake of ARVs for PMTCT. In Kenya, it was noted that women whose partners were involved had an increased adherence to Zidovudine than those whose partners were not involved; there was a low HIV acquisition among infants born to women whose partners attended the clinic and that partner attendance was associated with increased survival in HIV uninfected infants. The study suggests that MI may be an underutilized public Health Intervention (64).

2.5.3 Uptake of Human Immunodeficiency Virus Testing

Male involvement has a positive impact on a woman's willingness to undertake an HIV test. In DRC, secondary to MI in a PMTCT program, the percentage of women who accepted an HIV test increased from 72% to 99% (45), and of noteworthy in Zambia the rate of women who consented for HIV testing was higher, 96% among women counseled as a couple versus 79% in the group that was counseled without the partner (42). Similar results also were observed in Tanzania where women whose partners were involved in VCT and had an HIV test done went through VCT 12 times more than those women whose partners did not take an HIV test (65).Male Involvement can also be used as an opportunity to test the partner, which is an entry point into the preventive method (66).

2.5.4 Forum for Health Information Sharing

Women have argued that MI will increase the communication of health matters between couples. Among other things it will accord the man a chance to learn more aspects on the wife's condition (44). In South Africa, it was argued that the success of HIV prevention programmes was dependent on male involvement in the service, especially when men's interest were addressed within the programme (67). Including males in the programme elevates them to partners in the programme which will increase the

uptake of services by women because the PMTCT services would be supported by male partners. A review of studies indicated that it becomes difficult for a woman to seek medical care for herself and her children in instances where the husband is neither aware of her HIV status nor involved in the health care (58).

2.6 Strategies for Male Involvement

Despite the strong and supportive evidence on the positive impact of male involvement on health care use and adherence to health interventions by women, there have been no conclusive strategies made to include them (68, 69). The current way of measuring male involvement has been criticised as narrow. It needs further development from just men accompanying women for their appointments to include aspects of the natural social support by male partners since not all male partners would have the woman's health as a priority (17).

Literature presents several strategies that have been used to involve men in health services such as:

2.6.1 Introductory or Invite Letters

Introductory letters to the employers have been used to involve men in health services (70). Other form of invites have been through the spouses' ANC card (30, 49, 55) however it was noted that letters to partners have to be systematic and should aim at reaching all male partners for them to be effective (55). Nevertheless, it was further noted that men obliged to the formal invitations to the antenatal services (71). It is not surprising that there have been assertions on the need for partner notification and awareness policies in order to increase uptake of HIV testing (17).

2.6.2 Male –Friendly Environment

An understanding of the men's behavioural factors and masculine philosophies in the context they exist is likely to provide useful entry points in instances of gender inequality. Male Involvement requires creation of an environment where men can express freely their experience with HIV, eventually leading to positive change in men's attitudes and behavior(72). The success of programmes in patriarchal societies where men are decision makers on family matters relies greatly on consideration of gender relations in the programme (73). Additionally, it has been argued that such programmes ought to be within the community and not hospital based and need to consult the local men on the programme because masculinity is defined and developed at a community level (53). Clinic space offering privacy for male partners and male-friendly clinics has potential of increasing MI in PMTCT services. Accessibility of services was also seen to improve

or encourage MI (55). Clinics that open over the weekends and for longer hours have been advocated for in order to accommodate those that are working during the week days (30, 34, 43). Further to this, Byamugisha advocates for services that are geographically closer to the targeted people (49) and Falnes added that the services could further be streamlined for men or for pregnant couples only (46).

2.6.3 Male Education

Other strategies of improving MI are educating men on the importance of ANC and PMTCT (55). Tweheyo asserted that equipping males with information on antenatal care increases MI and a woman's use of that service (48). Respondents in an all-male Ugandan study suggested sensitizing men on ANC, PMTCT and the benefits of the services for them to take an active role in the services (49).

2.6.4 Couple Counseling

There is need for MI programmes to capitalise on the already existing programmes within the antenatal clinic such as couple counselling; this will offer HIV testing in adults who may not seek it (74). Studies in Kenya and Democratic Republic of Congo (DRC) have concluded that couple counselling is an acceptable strategy that can be used to increase male involvement in ANC (45, 74). Conversely, Falnes noted that in much as couple counselling is a good and presumably effective strategy, it may not achieve its intended purpose in settings such as in Sub Saharan Africa where males feel that antenatal clinics where PMTCT takes place is a woman's domain (46). Further to this, a study in Uganda stressed that a major obstacle with couple counselling is the responsibility that is placed on a woman to extend the invitation to the male partner who may be unwilling. This is further limited when she does not have the power to discuss such a sensitive issue and also the fear of potential adverse consequences from the partner (75).

2.6.5 Male inclusion in the Programme

Msellati et al propose a change on the term "prevention of Mother to Child Transmission" to "prevention of parents to child transmission" so that the mother is not solely portrayed as responsible for the transmission; this may promote MI in PMTCT services. Msellati et al further states that there is need for "mass campaigns" towards couples and communities for HIV testing for women and their partners (76). Reece et al further asserts that there is need to use leaders in communities to act as peer discussion leaders to facilitate discussions on MI in PMTCT (43).

2.6.7 Mass Media Campaigns

Health workers in Tanzania advocated for mass media as a means of male involvement in PMTCT. They suggested use of Television programmes in order to draw men's attention, family or couple seminars on PMTCT and also use of community or government leaders to advocate for PMTCT in their meetings (36). Further to this, Falnes proposes community mobilization in order to engage men in MI (46) and in agreement Skovdal asserts for the need for community involvement and consultation with local men in these programmes because masculinities are developed and negotiated at community level (53).

2.7 Literature Review of Studies done in Malawi

2.7.1. Description of Male Involvement in Maternal Health Services in Malawi

A study that looked at MI in Maternal health services in Mwanza, described MI in various ways such as, a) a program for Couple HIV testing and counselling where couples seek HIV testing through the PMTCT programme; b) a law enacted by the government whereby male partners are expected to accompany their partners in order for their partners to access antenatal care; c) a strategy for fast attendance to pregnant women in the antenatal clinic because women presenting for antenatal care with their partners are a priority in accessing care; d) unfair programme for pregnant women without partners as it highlights the absence of the partner; e) a foreign concept copied from western countries and an act of love within the couple. The study further observed a fragmented nature with the implementation of MI in maternal health in that it is not fully integrated in the service because it is associated with specific aspects such as the initial antenatal visit and HIV testing(77) .

2.7.2 Male Involvement in Prevention of Mother to Child Transmission of HIV services

In Malawi, just as in many Sub-Saharan countries, men are traditionally not encouraged to take part in pregnancy and childbirth issues; as a result of this, tradition health workers have not reinforced male involvement in care surrounding pregnancy. Consequently, this affects the level of MI in PMTCT as men try to conform to culturally correct practices (78, 79). Although cultural norms, especially in patriarchal societies, confer the decision making responsibility including decision on utilization of reproductive health services to men, they are rarely involved in PMTCT services. The lack of involvement is further aggravated by the inadequate knowledge that men have on the available services (80, 81). Owing to the patriarchal culture in Malawi, it has been asserted that women will adhere to decision made by a

male partner which raises the need for MI in PMTCT services in order to increase a woman's uptake of the service (81).

2.7.3 Rates of Male Involvement in Prevention of Mother to Child Transmission of HIV Services in Malawi

Rates of MI in PMTCT services have not been extensively measured however a non-randomised study done in Balaka noted that only 23% of the women in the study were accompanied by a partner for antenatal clinic care. Of those that were accompanied by their partners, 60% reported that the decision to take PMTCT services was enhanced by the partner's involvement in the service because they were counselled and tested as a couple. Furthermore, women reported that husbands reminded them to take Nevirapine, early reporting for labour and delivery, provision of information to health workers about the wife. On the other hand, women whose partners were absent during the PMTCT services expressed a concern over lack of couple counselling on HIV testing and risk reduction strategies (82). The women further indicated that their partners would have received information and counselling on condom use and infant feeding options directly from the health workers as opposed to getting information from the women (82).

Another study in Blantyre further showed that only 5.2% of the women were ever accompanied by their husband's for antenatal care despite the belief held by 71.3% of the married women in the study that their husband would accompany them for antenatal care (79). A study done in Zomba, in 2009 showed that there was a low efficiency and effectiveness in the PMTCT program that was done under routine program conditions. The study measured PMTCT effectiveness by using HIV-free survival at 18-20 months as an indicator and this was reported to be 66%. Additionally, the study showed that only 18% of the HIV infected mothers followed all the PMTCT Guidelines (83).

2.7.4 Challenges with Uptake of Prevention of Mother to Child Transmission of HIV Services

Currently the uptake of PMTCT of HIV services in Malawi has been faced with some challenges. One of the challenges with the uptake of PMTCT services has been resistance by the male partners in their female partners participating in the service thereby concluding that MI is key in Malawi where men are the decision makers in most households (26). A study done in Balaka reported that 21% of the women perceived that their husbands may prohibit them from taking an HIV test because men undermine

PMTCT services (82). It was noted that though the uptake of PMTCT has increased, men have not come forward for HIV testing through these services (7).

2.7.5 Barriers to Male Involvement in Prevention of Mother to Child Transmission of HIV in Malawi

A study conducted by Aarnio et al showed that the unwillingness of men to know their HIV status and the threat of HIV on their marriages compromised their involvement in the antenatal HIV counseling services.. The status of the marriage was also cited as a barrier to MI in that a weak marriage would not be conducive for MI as opposed to a strong marriage (41). Similarly, it was noted that barriers to male involvement in Lilongwe were a lack of perceived benefits of the service when one is healthy, lack of proper communication between partners and stigmatization fears (84). In addition, another study found that PMTCT was not considered a responsibility of the mother alone; they perceived it as a role of the husband and the community that needs a community's response. However, this perceived responsibility is not reflected in the current delivery of the services in the antenatal clinic where a client is a non- contributing partner but a cooperative recipient of care (15).

Like in other studies in Sub Saharan Africa, a study done in Blantyre and Balaka, Malawi, on the factors associated with low uptake of PMTCT service, reported that, women cited, among other factors, fear of the husband's reaction as a reason for not participating in the service even though they were eligible. It further indicated that some women reported experiencing abuse from their husbands' upon disclosure of one's HIV sero-positive status eventually resulting into divorce. Further to this, it was noted that rarely would a woman decide on taking up PMTCT services entirely on her own without prior consultation with the husband out of fear of negative consequences that may arise such as divorce (26, 80) .

2.7.6 Consequences of Lack of Male Involvement

The lack of MI has shown negative consequences around Malawi. In Lilongwe, it was reported that some women dropped out of a PMTCT programme upon husband's request because he doubted the HIV positive results of the wife (15). Similarly, a study done in Thyolo, among other reasons, women also cited lack of partner support as a reason for loss to follow up in a PMTCT programme especially in those men who did not want to take an HIV test. Furthermore the study revealed that most men would not accept an invite from the wife to attend PMTCT because it would affect their masculinity if they accept and also because the programme takes place in women's domain. Consequentially in this area

there were only a few women and children taking ARVs, because of their husband's opposition to HIV testing consequently failing to access ARVs (14).

Secondary to lack of MI in PMTCT services in urban Blantyre, women opted to delay or defer a test until a husband has approved of it. The study suggested male participation as one of the measures of reducing number of women refusing an HIV test (16). This was further confirmed in Balaka where a study on factors associated with use of PMTCT services by young adults showed that the lack of support from their male partners negatively affected the woman's usage of the PMTCT service. The study further showed that some participants believed that their partners would not allow them to use the PMTCT service (82).

Culturally, women in Malawi have a limited voice on issues concerning their life. They are not expected to make higher or major decisions as these are left for the man to make in his capacity as the decision maker in the society. This notion is rooted at a very young age where society tends to value boys more than girls. The sex education received by boys and girls is different implying variation in the expectation from each gender; girls are asked to practice abstinence while boys are told to be careful with whom they have sexual relations with. The variation in the education received promotes the gender based differences which later negatively impacts on the woman's ability to negotiate for safer sex or make decisions for own health. Furthermore the dynamics of relationships between the two genders limits the woman's ability to protect herself from HIV and AIDS (85) and the lack of MI can be argued to enhance this culture.

2.7.7 Strategies Used in Malawi

A study done in the Southern part of Malawi on the strategies that are used to invite men into the general Maternal Health services, highlighted four main strategies currently being used and these are;

a. Health care provider initiative where the health care provider takes the initiative in asking the woman to have the partner involved by informing the woman the benefits of such.
b. Partner notification, although this strategy has been criticised that it reduces men as not beneficiaries of the service.
c. Couple initiative, where couples make a decision that both partners, wife and husband, will be involved although this was only common among the more elite couples.
d. Community mobilization, in which traditional leaders are involved as advocates for male involvement in maternal health services. Community mobilization occurred at different

levels for instance through male peer initiative, use of incentives for the area that registered more men in MHC service and community sensitization.

Although there were strategies identified, the study also noted that some of the strategies were not sustainable because they depended on several assumptions such as willingness of a woman to extend the invite to the husband and availability of incentives for men to be involved in MHC. The study concluded that there is need to identify lasting strategies for MI that will not be dependent on incentives, invitation or coercion (86).

Following a study on PMTCT in Thyolo, the hospital management put up the following measures to overcome the low levels of MI in reproductive health care, a) asking women to extend invite to their partners for couple counselling and b) exemption from the queue for the men who accompanied their partners for antenatal clinical care. These strategies were reinforced through consultative meetings with men, women, village headmen and faith based leaders. These strategies resulted into 10% of women reporting at the health facility in the company of their partners (81).

Fast attendance to a pregnant woman who reports with a partner for antenatal care is another strategy for male Involvement in Maternal Health services. However this strategy was criticised for marginalizing women without partners and highlighted them as women in a non-loving relationship or those who got pregnant outside of marriage (77).

2.8 Conceptual Framework- PRECEDE-PROCEED Model of Health Program Planning and Evaluation

The study will be guided by the PRECEDE-PROCEED Model of Health Program Planning and Evaluation. PRECEDE-PROCEED Model is a planning model. It has two components a) PRECEDE part and b) PROCEED part. PRECEDE stands for **P**redisposing, **R**einforcing and **E**nabling **C**onstructs in **E**ducational/Environmental **D**iagnosis and **E**valuation. The PRECEDE part outlines the steps that take place before an intervention for a problem is implemented. PROCEED stands for **P**olicy, **R**egulations and **O**rganization **C**onstructs in **E**ducational and **E**nvironmental **D**evelopment. The PROCEED part outlines the steps and procedures in carrying out or implementing the suggested intervention. It is a logical model because it outlines steps in planning, implementing and evaluating a health programme. It links the causes of a problem to the implemented intervention for the problem. Researchers' using this model develop strategies from a precise understanding of the problem under study because this model builds on the premise that understanding of the problem should precede the intervention to be implemented as a solution to the problem (87, 88). The model has 8 steps; four steps are in the Precede part and the other four are in the Proceed part of the model.

The diagram below presents the authors adaptation of the model the study

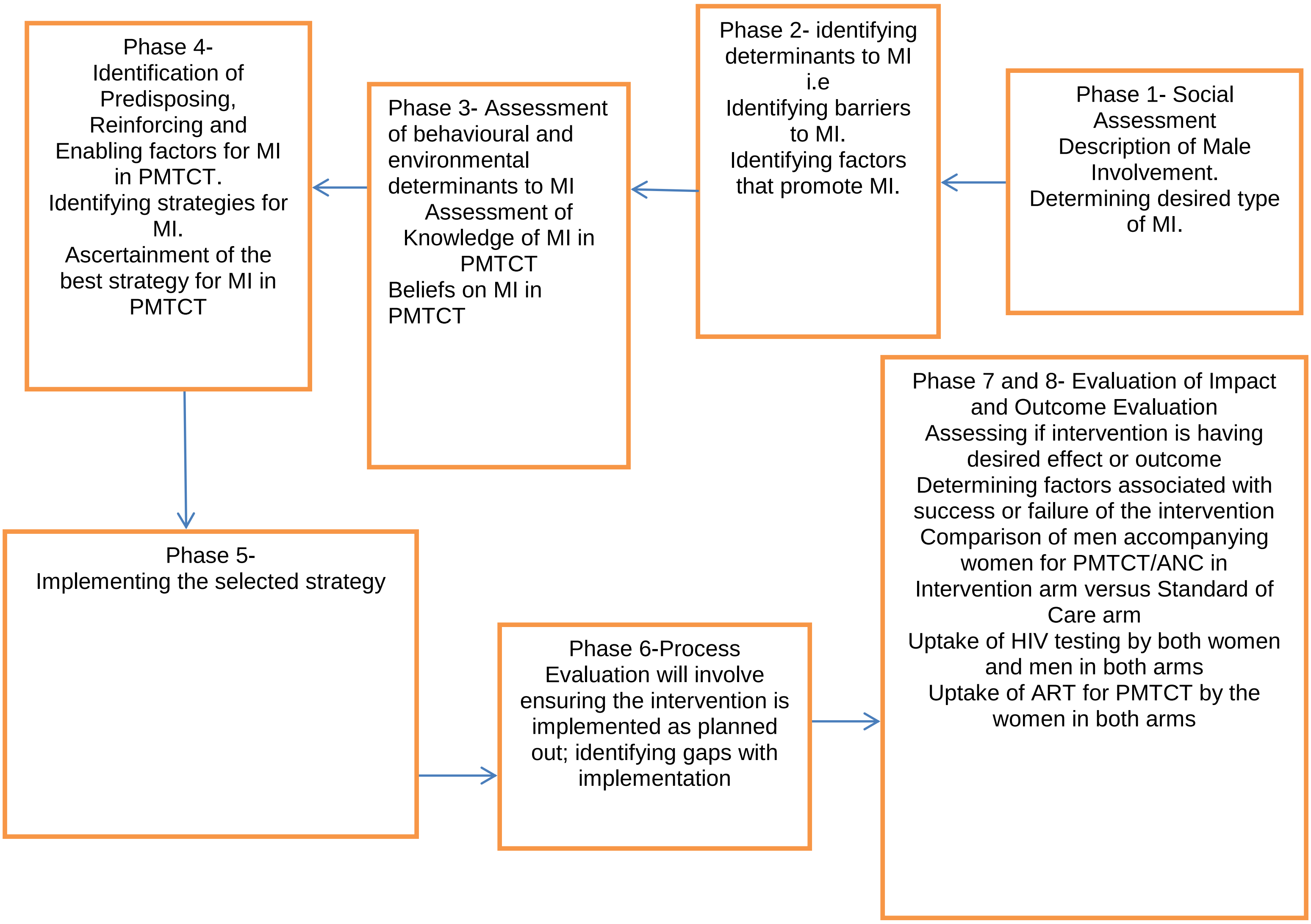

Figure 1 - The Authors' application of the PRECEDE PROCEED Model to the study

Selection of this model is secondary to the flexibility of the model. The steps in the model are not rigid; researchers can modify them to suit the particular topic under study. It allows for a participatory approach because it allows the researcher to identify factors pertaining to the issue under study together with the people affected and who are also service users. It also assists in developing a solution that is evidence based for the problem under study.

This model is suitable for the study because it allows the researcher to make a description of MI in PMTCT; identify the factors that hinder or promote MI and the strategies of involving male partners. It further allows for testing of the identified strategy in order to determine feasibility and effectiveness of

MI. This model follows a logical and structural way which assists with determination of progress as one develops and implements MI in PMTCT services. It also allows for a multisectoral approach in solving a problem because it considers various factors that could influence the problem.

Details regarding the intervention, identification, implementation and evaluation of the strategy are discussed in Chapter 3 which discusses the Methods for the study.

2.9 Conclusion of Literature review

The literature review has focussed largely on the description of Male involvement. It has also delved to explain both the barriers and the benefits of MI in PMTCT. There has been consistent literature on the positive effects of MI on uptake of PMTCT services. Literature in Malawi has not revealed strategies that are in place that have been tested for effectiveness in Malawi. To capitalise on MI as catalyst for PMTCT, it is imperative that a strategy that has been tested be put into use for PMTCT use. Therefore, this chapter has presented the basis for the next chapter which will outline the method of identifying such a strategy.

CHAPTER 3 METHODS: INTERVENTION PHASE

3.1 Introduction

This chapter presents the methods that will be used in the intervention phase of the study on MI in PMTCT. The chapter will cover the study design, the study setting, study population, projected study period and timeline, sample size, data collection method, statistical considerations, planned presentation of results, planned dissemination of results, ethical considerations, possible constraints, requirements, budgetary estimates and the budget justification. This part follows after analysis of formative phase results as reported in version 2.0 of the proposal. The formative phase informed the intervention that will be used in the intervention phase of the study.

3.2 Study Design: Randomised Control Trial

The study will be a randomized open label trial among pregnant women in the antenatal clinics to determine the feasibility and effectiveness of an invitation card as a MI strategy in PMTCT services on the uptake of the services. Randomization will be stratified by health centre. Pregnant women will be enrolled at any time point in pregnancy, until 30 weeks gestation. The study will have two arms, Arm A and Arm B. Arm A will be the intervention arm where women will be randomized to use of an invitation card as a strategy for MI in PMTCT of HIV while in Arm B, the non-intervention arm, women will not use the invitation card. The outcome measures for the study will be on the uptake of PMTCT services defined as follows

- The number of pregnant women accompanied by their husband's or partners at week 2 or 6 of the study secondary to the strategy
- The number of men accepting HIV testing
- The number of women taking up the PMTCT services such as HIV testing, ARVs for PMTCT, Infant feeding Options
- The number of women adhering to treatment ; using pill count and keeping appointment dates

The information will be collected from both the intervention and non-intervention arm.

Randomization ensures that factors that form the baseline for testing statistical significance between the two groups' are distributed equally between the groups thereby balancing the distribution of factors such as age, sex and other baseline factors that could be potential confounders in a study (89).

3.3 Recruitment and Enrollment Procedures

At the antenatal visit, a woman will be informed about the study. A health education talk is generally given at the antenatal clinic and PMTCT of HIV and general antenatal care are discussed. During the antenatal talk, the study will be introduced and the study details will be shared during the antenatal consultation in the cubicles within the Health Centre. Consecutively presenting women will be approached to solicit interest in the study. If interested, they will be referred to the cubicle where a detailed discussion on the study and all consenting procedures will be conducted. Consent will be individually sought from all eligible women (see Appendix I). Consenting will be done by trained study nurses and will be written consent. If a participant is illiterate then participant will be asked to thumbprint the consent form in the presence of a literate witness. The entire consenting process for an illiterate potential participant will be done in the presence of a literate witness. The witness will be asked to sign and date the consent form to attest the accuracy of information relayed to the potential participant and also that the potential participant understood the information and that the consenting process was non-coercive.

Eligibility screening will be conducted by study nurses and details of screening will be captured in a screening log. Each antenatal woman screened will be assigned a screening number. The screening log will be kept at the clinic for the entire duration of recruitment and will be transferred to College of Medicine after recruitment is over. The log will give more information on the number of women approached for the study, those who were eligible and enrolled and also reasons for non-eligibility for those not enrolled in the study.

Gestational age, being exclusion criteria, will be estimated by using both fundal height as measured by the midwife and last menstrual period (LNMP) dates as provided by the woman. Women who are **>30** weeks will be ineligible for entry into the study and will be referred for standard of care. HIV status is not a criterion for eligibility. Two consent forms will be filled out for each participant; one for the file and one will be given to the participant.

A computer generated randomization list will be used to sequentially assign the women between the two arms in the two Health Centres. Randomization will be stratified by health centre to balance for randomizations in each health centre using permuted blocks with variable block sizes. The generated randomization list will be securely kept in a locked cabinet at the College of Medicine. Participants will be randomized within block after block until the sample size is realized. A separate randomization list will be prepared for each health centre. Opaque sealed envelopes, numbered sequentially and containing a slip of paper with the randomization ID will be used in order to avoid bias during assignment of IDs, with an allocation ration of 1:1. After obtaining informed consent, and deemed eligible, the next envelope in order will be opened to reveal the participant's study assignment. The

envelope will be opened in front of a participant. The participant will then be informed immediately about the study assignment as per slip in the envelope. If the potential participant is ineligible, as per inclusion and exclusion checklist, then the potential participant will be referred back for standard antenatal care. At all the study visits, there will be interviews done to both the women and their partners' on the uptake of PMTCT services. The study visits will be at entry, study week 2 and 6 and will be aligned or scheduled as per participant's antenatal schedule. In the event that a male partner for reports to the clinic, informed consent will be obtained from them after assessing eligibility following the male partner informed consent (See Appendix II).

The Intervention study design is summarized in Figure 2 below

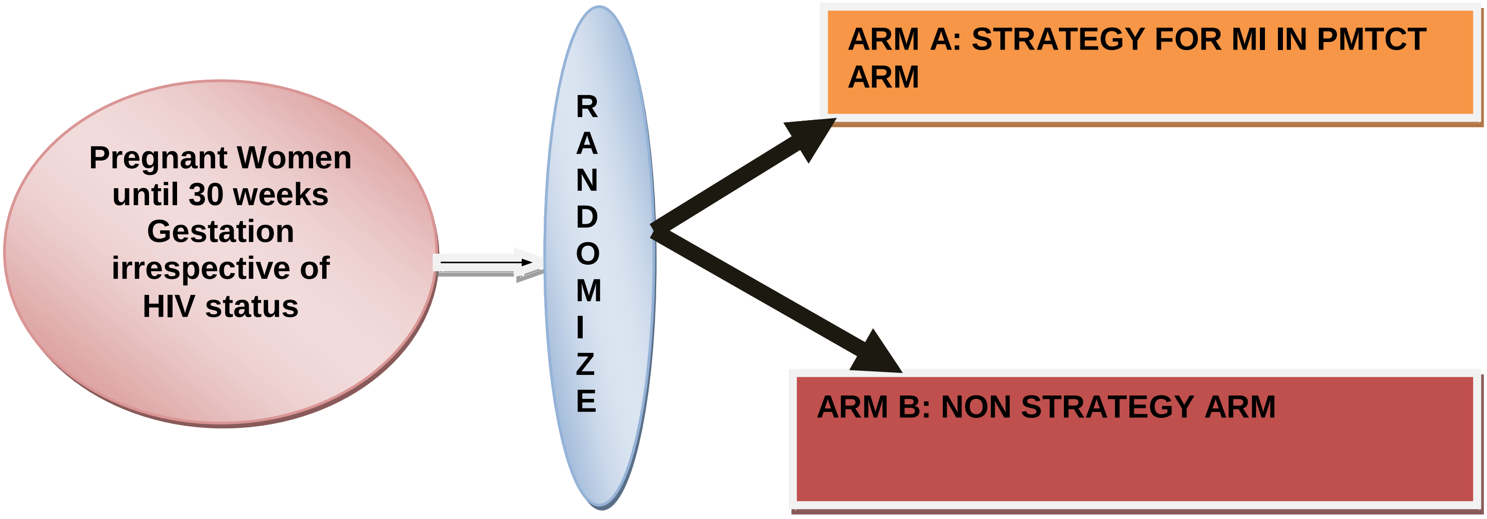

Figure 2: Randomization into the two arms in each Health Centre

3.4 Study Place and Setting

The study will be done in Blantyre district in the Southern part of Malawi. Blantyre is a commercial city in the southern region. According to the population census of 2008 Blantyre district has a population of 1,001, 984 consisting of 501,000 males and 500, 984 females (90). Blantyre has about 32 Health centres. Some are managed by Blantyre District Health Office while some are co-managed with Blantyre City Assembly. According to records at the Blantyre DHO's Office, in 2011, the reported percentage of HIV infected pregnant women was 10.4%. The specific health centres that will be used are South Lunzu Health Centre and Mpemba Health Centre.

South Lunzu Health Centre is 11 km from Blantyre city centre, and located on the south eastern part of Blantyre District. It serves a semi urban population. According to the Blantyre District, Health Office, the catchment population for SLHC in 2008-2009 was estimated at 77, 403 with about 18, 101 households. The expected pregnancies and deliveries in the same period were 3870. South Lunzu Health Centre provides the following services, Out Patient, Voluntary Counselling and Testing, Family Planning, Directly Observed Treatment for Tuberculosis, Maternity services which have Antenatal care, Labour and delivery and Postpartum services. The Health Centre has the following categories of health personnel, medical assistants, nurse/midwives, community health nurse midwife technicians and health surveillance assistants. On a monthly basis, the SLHC registers about 200 new antenatal women South Lunzu Health Centre runs antenatal clinics on Mondays for initial visit and on Tuesdays and Thursdays for subsequent visits.

Mpemba health Centre is situated on the southern part of Blantyre district and is about 10km from Blantyre City Centre. It serves a semi urban population. According to the Blantyre District, Health Office, the catchment population for MHC in 2008-2009 was estimated at 38, 251 with about 9, 307 households. The expected pregnancies and deliveries in the same period were 1913. Mpemba Health Centre provides the following services, Out Patient, Voluntary Counselling and Testing, Family Planning, Directly Observed Treatment for Tuberculosis, Maternity services which have Antenatal care, Labour and delivery and Postpartum services. The Health Centre has the following category of health personnel Clinicians, Nurses and Health Surveillance Assistants.
On a monthly basis, the MHC registers about 100-150 new antenatal women and runs antenatal clinics on Mondays for initial visit and on Tuesdays and Thursdays for subsequent visits .The PMTCT Services are offered within the Antenatal services and include HIV Testing and counselling, provision of ARVs and cotrimoxazole, Infant feeding counselling
Most pregnant women in Malawi report for antenatal care in their second trimester with about 92% reporting at that time (13). Although pregnant women are encouraged to invite their partners for antenatal care and PMTCT services, a few men under 2% show up at the clinic. The rate of MI in PMTCT of HIV infection, as reported by health care workers from both health centres, is very low.

3.5 Study Population

The study population comprises of women utilizing the antenatal health services and men who are partners of the women who are pregnant and using the health facilities within the study area.

3.6 Sample Size and Accrual

The primary measure is the proportion of pregnant women who report to the study clinic with their partners, for PMTCT services following use of the strategy after 2 visits. The estimated uptake of a strategy for MI in PMTCT is estimated at 16% with use of a strategy. The estimate is based on a Ugandan study where male partners were invited for antenatal services/PMTCT services using an invitation letter; the study showed that 16% of the pregnant women in the intervention arm were accompanied by their partners at their next scheduled antenatal visit while 14% of the pregnant women in the arm that used an information sheet about antenatal services on offer were accompanied by their partners as well (91). Based on the Ugandan Study, the assumption made is that Male involvement in PMTCT services will increase from 2% (without intervention) to 12% (with intervention) with 90% power and 5% significance level, the sample size calculated in stata is 208 in each arm and after adjusting for a 10% attrition rate the final sample size will be 231 in each arm giving a total of 462. The decision to use such an effect size is based on that to date there have not been trials that have compared strategies in Malawi as well as the lack of stipulated strategies available such that such a difference would be deemed enough as a starting point for MI in PMTCT of HIV services.

3.7 Selection of Participants: Inclusion and Exclusion Criteria

Inclusion Criteria:

1. Pregnant women, until 30 weeks gestation. Gestation period is limited to 30 weeks to allow room for adequate evaluation of the strategy as they report for antenatal care. Ascertainment of gestational age will be through fundal height assessments and or calculating from the Last Normal Menstrual Period date.
2. Married pregnant women aged 16 years or older will be eligible because according to Malawian law, they are considered emancipated and are able to sign the consent form for themselves without parental consent.
3. Pregnant women must be attending antenatal care without a spouse.
4. Planning to attend the antenatal care at the study clinic.
5. Ability and willingness to provide informed consent before study participation
6. Pregnant women with a male partner responsible for the current pregnancy
7. Women should be willing to pass on the “invitation card” to the partner.

Exclusion Criteria:

1. Postpartum women- Postpartum women will be excluded to avoid running the study for a long duration and also because there are only a few postpartum visits that postnatal women attend which may create a potential for loss to follow up in postpartum care.
2. Pregnant women with gestation over 30 weeks. Gestation above 30 weeks will not offer adequate time to follow up to assess uptake of the intervention.
3. Unwillingness to join the study.
4. Unwillingness to comply with study visits.
5. Women without a male partner.
6. Women who attend antenatal care with their spouse.

Note: HIV status is not a criterion

Procedures during Study Visits

The evaluations are as follows:

Table 1- Evaluations during study visits

**Non Laboratory Data**	**Laboratory Data**
• Maternal and Partner Demographics<br>• Obstetric History<br>• Sexual History<br>• Attendance to PMTCT together<br>• Husband's/Partners' support on PMTCT uptake<br>• Husband/Partners' /Woman's view over the strategy	• Confirmation of status through Health Passport books Referral/Offering of HIV Test as per National Guidelines

3.8 Data Collection

Structured questionnaires will be administered for data collection from women and their partners in the two study arms. The interviews will be done in a private room and will be done in Chichewa. Data collected will be on socio demographic details, obstetric and sexual history, uptake of PMTCT services and Male involvement in PMTCT services following administration of an invitation card.

3.8.1 Validity and Reliability of Data Collection Tools

3.8.1.1 Validity

Validity of the data collection instruments will be measured in this way:

Content Validity

The information on the questionnaires will be compared with the objectives to validate if the questionnaires are representative of the possible questions one can ask under the objectives. Since content validity is based on judgement (92) the questionnaire will be circulated to experts in the area to judge the contents of the questionnaire and determine the relevance of the items.

Face Validity

The questionnaires will be looked at to assess if they look to be measuring the right content just at face value (92). This assessment will be done by other researchers.

3.8.1.2 Reliability

Reliability refers to the consistency of the measuring instrument over time. It is also the application of the same procedures and arriving at the same results at different times. The data collection tools will have some questions that are the same but will be phrased in a different way; this will assist in assessing whether the participants give the same answers which will verify reliability of the tools. Reliability will also be increased in the study by ensuring that a question is only asking about one concept other than multiple questions. In instances where a response is unclear, the researcher will ask for clarification to ensure accuracy (93). The data collection tools will be piloted on a few participants who will not be part of the sample size to determine reliability of the tools. The results will be compared for consistency after initial and subsequent administration which will confirm reliability if responses remain consistent (92).

3.9 Data Management

Research assistants will be trained in data collection using the Case Report Forms (CRFs). Key pieces of information such as information of male uptake of PMTCT services will be reviewed and verified at each visit by the Principal Investigator. A coding guide will be developed prior to data entry. Data will be checked for consistency and completeness and double entered in a computerized database. Each participant will have her own file with the partners file attached to it with all the CRFs in it and progress notes where

necessary. The Investigator and Data Manager will run regular quality checks and any inconsistencies will be addressed immediately. Adequate back up system will be used. Microsoft Access/Epi Info will be used for data management and the computer for data management is protected against computer viruses by antiviral software.

At the end of the study when all data cleaning procedures have been finalized, the database will be locked. All study documents will be stored in secure and confidential conditions. On all study documents except for the consent form, participants will be identified with a unique participant number and not by name.

3.10 Data Analysis

Intent to treat analysis will be employed. Intent to treat analysis implies analyzing data for the primary outcome as per initial assignment regardless of whether they complete the study or not. Intent to treat analysis avoids the effects that may result from participant withdraw or drop outs from the study eventually affecting the randomization to the intervention arm. The proportion of women who return to the clinics with their partners will be compared among the two arms and among the two health centres. The secondary outcomes will measure the number of male partners who report to the clinic and take an HIV test and also the uptake of PMTCT services in the two arms. Data analysis will take stratification into account.

Descriptive statistics will be used to describe and synthesize the socio demographic data in the study. The socio demographic characteristics of the participants in the intervention arm (arm using the strategy) and non-intervention arm (arm not using the strategy or arm following standard of care) will be compared using independent t-test for continuous variables and a Chi-square for the categorical variables.

Inferential statistics will be used to draw conclusions about the population under study. It is being hypothesized that the rate / proportion of male uptake of PMTCT between the two arms understudy is the same and the alternative hypothesis is that the rate of male uptake in PMTCT in two arms is not the same.

$$H_o : \pi_1 = \pi_2$$
$$\pi_1 - \pi_2 = 0$$

$$H_a : \pi_1 \neq \pi_2$$
$$\pi_1 - \pi_2 \neq 0$$

Where π_1 is the proportion in standard arm π_2 is the proportion in the intervention arm

A standardized z-test (Normal Test) will be used to test the hypothesis as a result of the central limit theorem (asymptotic) where Confidence intervals are derived from calculations based on the normal distribution. Pearson's chi-square test would be used to test for a difference in proportions. All statistics (Odds ratios) will be estimated with 95% Confidence Intervals to assess the strength and direction of association of demographic and behavior characteristics of MI in PMTCT services.

Hypothesis testing for comparing proportions

A standard normal distribution for proportions will be used because of the Central Limit Theorem.

Assumptions in the study

- Independent random samples receive the two treatments
- Sample size is large enough to allow use of the standard normal distribution, sample size>30

Decision Rule

- Reject H_o if $p < 0.05$

Adjustment for potential confounders will be done through logistic regression.

3.11 Study Period

The estimated study period is as follows:

Ethical review Process- This will take a minimum of 1 month. The amended protocol has to be approved by College of Medicine Research and Ethics Committee (COMREC) prior to implementation.

Training- There will be multiple trainings running for a week at several intervals. There will be a minimum of 3 training sessions prior to study implementation-

Data Collection- Data will be collected over a period of ± 1 year

Data analysis- This will take 4-6 months

Report Preparation- This will take 4-6 months

Dissemination of Results- The process may take 6-9months.

3.12 Results Presentation

The results will be presented in Tables and Charts. There will also be a narrative summary of the results.

3.13 Dissemination of Results

The report will be submitted to the Department of Community Health at College of Medicine as a requirement for the fulfillment of a Philosophical degree. The information obtained will be summarised in manuscripts, and will be submitted to peer reviewed journals. In addition, Policy Briefs and dissemination of findings will be held with the Ministry of Health, Blantyre City Assembly and Blantyre District Health Office. The results will be used as needed to inform local policy on MI in PMTCT which will eventually assist in the incorporation of males in the service. The results will also be disseminated through conferences locally or internationally.
The results will also be disseminated through Seminar workshops within the Colleges of Medicine and Nursing.

3.14 Ethical Considerations

Equipoise
Equipoise exists to support this randomized trial. At this point, it is not known whether there is a strategy that is better and more relevant for MI in PMTCT Services in Blantyre, Malawi. Similarly, it is also not know whether provider initiated through letters or other form of invites or male initiated involvement as is the current practice in some instances is an effective strategy. By testing a strategy that has been identified by the end- users, we hope to establish an effective and feasible strategy for MI in PMTCT services which may increase the uptake of the service by pregnant women and their partners.

Informed Consent

This proposal, the informed consent forms, participant recruitment and education materials and any subsequent modifications will be reviewed and approved by COMREC before implementation. Consent forms will be translated and used in Chichewa. The informed consent form will be either read or be given to the participant to read. Written informed consent will be obtained from a potential participant before any study related procedures are done. A literate participant will be asked to sign the consent form once she/he has read the form.   An impartial witness will be present when obtaining consent from

an illiterate participant. An illiterate participant will be asked to thumbprint the consent form after it has been read to him or her. The informed consent will describe the purpose of the study, the outline and the risks and benefits of participation, the duration of the study, procedures to be followed, expected nature of participation from participants. A copy of the consent form will be offered to the participant to take home. When the partner reports to the clinic for study activities, consent will also be obtained from him/her before any study procedures.

Risks

Although every effort will be made to protect confidentiality and privacy, other people may learn of the participants' involvement with the study. Social harm may result if people suspect that the rationale behind partner involvement in PMTCT programme is associated with HIV infection. Another potential risk is when couples undergo an HIV test together and have sero discordant HIV results. This may cause social problems between the partners. However, counselling on HIV Pre and Post-test counselling is done by trained counsellors and this will potentially minimise the problem and where necessary participants will be referred for additional couple counselling.

It is also possible that participants may find participating in this study stressful or uncomfortable.

Benefits

The benefit in participating in the study is that male partners will be referred for an HIV test. Information learned from this study may be used in involving male partners in the PMTCT services of their partners.

Incentives

There will be no incentives paid out for participating in the study, however participants' transport costs will be reimbursed.

Participant Confidentiality

All forms, reports, and other records will be identified only by a coded number to maintain subject confidentiality within the research record. All records will be kept in a secured area with limited access. All computer entries will be done with coded numbers only.

All records that contain names or other personal identifiers such as consent forms, locator forms, log books and appointment books will be stored separately from the study records. The data base for the study will be secured with a password-protected access system.

The participants will be assured that their responses and views will not be shared with anyone except with authority bodies such as COMREC and MOH if necessary.

Privacy

All study interviews at the clinic will be conducted in a private room or office. The researcher will only collect data deemed necessary for the study and no data of private in nature than necessary will be collected. All women who refuse to participate in the study will be assured that their normal medical care will not be affected in any way.

Minimizing Coercion

All women and men, irrespective of their participation in the study, will be able to receive all their regular care at the health centre. The study staff will work closely with the Health Care workers to ensure that individuals who are not participating in the study access their care without problems.

Serodiscordant Issues

Couples needing HIV testing as a couple will be referred appropriately for couple counseling and testing as per referral system within the Health Centre. If need be, couples will be referred to other stakeholders who are working with couples on HIV Testing within Blantyre such as MACRO and Johns Hopkins Research Project for further counseling and management.

Strategy for the RCT

The strategy that will be identified in the Qualitative Phase will be submitted in a Letter of Amendment to COMREC for review and approval before implementation of the Quantitative Phase of the study.

3.15 Possible Constraints

The challenges envisioned by the researcher are a) longer recruitment period and duration of follow up during the intervention phase. The possible ways of addressing identified problems will be as follows; In order to reduce losses to follow up during the RCT, all possible measures such as use of appointment cards will be in place to remind participants of their appointment dates. Appointment cards will be filled out at every visit with the next visit date. All participants will be provided with a business card that has the details of the Investigator such as a telephone number for them to call if needed.

3.16 Study Requirements

The study will require the following:

Personnel

Study Coordinator/Investigator (1)- She will oversee the overall implementation of the study from preparation, ethical review submission, training of staff on the protocol and management of the protocol until study closure.

Data Manager (1)- He/She will be responsible for data management, quality checks on data and training of staff on completion of Case Report Forms or questionnaires.

Research Assistants (Nurses-2) - They will be responsible for administering motivational talk on the study, obtaining informed consent from potential participants, eligibility screening, administering the questionnaires in order to collect data.

Training

Interviewers Training- There will be training on the Protocol and its requirements such as obtaining informed consent, data collection and review of HIV testing and counselling. The training will be done after Ethical Committee/IRB approval. The Investigator will conduct the trainings. Additional training on Couple Counselling will be provided to the Nurses working on the trial by a facilitator from Johns Hopkins Project.

The proposed training is necessary to achieve uniform understanding of the protocol prior to implementation and also guarantee uniform data collection.

Stationery

The study will require stationery supplies such as paper for printing consent forms, questionnaires, reports and other administrative needs. There will also be need for a printer, toner, folders and binders for participant charts and regulatory binders, notebooks and hard covers for weekly reports on the study progress and other miscellaneous stationery such as markers, pens, papers, stick pads and staples. The study will also need a computer for data management purposes.

Transport

The study will require a dedicated vehicle for administrative needs of the study.

Space

The study will utilise existing structures within the Clinics

3.17 Budgetary Estimates

MI in PMTCT ESTIMATED BUDGET Jan- Dec 2013 BLANTYRE MALAWI				
**Personnel**				
		**Effort**	**Monthly Stipend**	**1 Year**
L. Nyondo Mipando	Investigator	100%	$ 1,500	$ 18,000
Research Assistant	RA	100%	$ 250	$ 3,000
Research Assistant	RA	100%	$ 250	$ 3,000
TBD	Tracer	100%	$ 75	$ 900
TBD	Data Manager	100%	300	$ 3,600
**Subtotal**				**$ 28,500**
**Clinic Supplies**				
**Supplies for Visits**	**$ 3.00**	**1350**	**$ 4,050.00**	
**Misc. supplies**			**$ 150.00**	
**Transport and refreshments**	**$ 5.00**	**1800**	**$ 9,000.00**	
**Subtotal**			**$ 13,200.00**	
**Tuition**				
**COM Fees**	**$ 2,125.00**	**1**	**$ 2,125.00**	
**Vehicle Hire**	**$ 200.00**	**12**	**$ 2,400.00**	
**SubTotal**			**$ 4,525.00**	
**Travel**				
**International Conference**	**$ 5,500.00**	**1**	**$ 5,500.00**	
**Local Conference**	**$ 2,000.00**	**1**	**$ 2,000.00**	
**Total**			**$ 7,500.00**	
**Office Supplies**				
**toner**	**$ 142.86**	**4**	**$ 571.43**	
**Paper**	**$ 7.14**	**24**	**$ 171.43**	

Folders	$ 10.00	80	$ 800.00	
Misc Stationery	$ 200.00	1	$ 200.00	
Fuel	$ 105.00	12	$ 1,260.00	
Hard Disk	$ 100.00	1	$ 100.00	
Microsoft Office	$ 160.00	1	$ 160.00	
Referencing Software	$ 330.00	1	$ 330.00	
Communication	$ 35.71	12	$ 428.57	
Computer	$ 850.00	1	$ 850.00	
Printer	$ 357.14	1	$ 357.14	
SubTotal			$ 5,228.57	
Training				
	$ 200.00	3	$ 600.00	
SubTotal			$ 600.00	
Other Expenses				
TOTAL BUDGET	10%	$ 5,547.86		
COMREC F&A				
Summary of Costs				
Personnel	$ 28,500.00			
Clinic Costs	$ 13,200.00			
Tuition	$ 4,525.00			
Training	$ 600.00			
Office Supplies	$ 5,228.57			
Travel	$ 7,500			
Total Budget	$ 59,553.57	$ 55,028.57		

3.18 Justification of the Budget

Personnel

This cost will cover for the remuneration for the following; Investigator, Data Manager, 2 Research Assistants and a tracer, who will work on the study. The Team will be responsible for implementing the

protocol, which will mainly involve screening of potential participants, recruitment, community liaison activities, administering questionnaires during follow up visits and data entry.

Study Clinic Supplies

This cost covers costs for the necessary clinic supplies that the Research Assistants may require as they conduct the study such as aprons, gloves other supplies as needed. This cost also covers participant's transport reimbursements and refreshments while at the study clinic. The specific calculation under this cost has been revised as follows:

Sample size is about 462 pregnant women and 231 male partners, giving a total of 693 and the projected number of visits is 3 for women and 2 for men with transport reimbursement and refreshments at 5 USD per participant per visit.

Office Supplies/ Other Expenses

These items are necessary for the daily administrative operations at the Clinics and Offices throughout the project. This cost will include Paper for Questionnaires and Consent forms, Printer and toner, Folders and other stationery as needed. The cost also includes costs for data management supplies such as, CDs, external hard disks for backup storage of the data, hardware and software and other computer accessories. The cost will also cover for the purchase of 1 Laptop computers that will be used for this study.

This cost will also cover communication costs; there will be a need for constant communication between the clinics and the participants/investigator. Communication costs will also cover internet connectivity charges, costs for photocopying the questionnaires and the consent forms and fuel for local running in the clinic.

Training and Community Activities

This cost will cover the training costs. The research staff will have to be trained on the Protocol prior to implementation. The training costs will cover for the cost of material used for training packages such as paper or printing the training material, toner for the printer, pens and pencils, folders and also cost for refreshments during the training.

Indirect Costs- F & A

This cost will cover for the 10 % F & A cost for the Malawi College of Medicine.

Tuition

This cost covers the tuition for the investigator payable to the College of Medicine for one year.

Travel

This cost covers travel costs plus incidentals for international and local conferences.

BIBLIOGRAPHY

1. UNAIDS. UNAIDS Report on the Global AIDS Epidemic, Geneva. 2010.
2. UNAIDS. World AIDS Day Report. 2011.
3. WHO. PMTCT strategic vision 2010–2015 : preventing mother-to-child transmission of HIV to reach the UNGASS and Millennium Development Goals.2010 14 April 2012. Available from: http://www.who.int/hiv/pub/mtct/strategic_vision.pdf.
4. Malawi NSO. Malawi Demographic and Health Survey Zomba, Malawi: NSO; 2010.
5. NAC. Malawi HIV and AIDS Monitoring and Evaluation Report:2008-2009, UNGASS Country Progress Report
Lilongwe2010.
6. USAID. HIV/AIDS Health Profile. Lilongwe2010.
7. MOH. Prevention of Mother to Child Transmission of HIV Guidelines. Second Edition ed. Lilongwe: Ministry of Health; 2008.
8. MOH. Quarterly HIV Programme Report. LilongweJanuary- March 2010.
9. WHO, UNICEF, UNAIDS. Towards Universal Access , Scaling up priority HIV/AIDS interventions in the health sector. World Health Organisation; 2010.
10. WHO, UNAIDS, UNICEF. GLOBAL HIV/AIDS RESPONSE: Epidemic update and health sector progress towards Universal Access 2011 Progress Report. World Health Organisation; 2011.
11. UNICEF. Children and AIDS: Fifth Stocktaking Report2010. Available from: http://www.unicef.org/publications/files/Children_and_AIDS-Fifth_Stocktaking_Report_2010_EN.pdf.
12. MOH. Intergrated HIV program Report. LilongweOctober-December 2011.
13. MOH. Intergrated HIV Program Report, January-March 2012. In: MOH, editor. Lilongwe2012.
14. Bwirire LD, Fitzgerald M, Zachariah R, Chikafa V, Massaquoi M, Moens M, et al. Reasons for loss to follow-up among mothers registered in a prevention-of-mother-to-child transmission program in rural Malawi. Transactions of the Royal Society of Tropical Medicine and Hygiene. 2008;102(12):1195-200. Epub 2008/05/20.
15. Chinkonde JR, Sundby J, Martinson F. The prevention of mother-to-child HIV transmission programme in Lilongwe, Malawi: why do so many women drop out. Reproductive Health Matters. 2009;17(33):143-51.
16. Nyondo Mipando L, Nkanaunena K, Seyama L, Kumwenda N. Barriers to HIV Testing in Prevention of Mother to Child Transmission Services in Blantye. National HIV and AIDS Research and Best Practices Lilongwe: NAC; 2011. p. 22.
17. Maman S, Mbwambo J, Hogan NM, Kilonzo GP, Sweat M. Women's barriers to HIV-1 testing and disclosure: challenges for HIV-1 voluntary counselling and testing. AIDS Care. 2001;13(5):595-603. Epub 2001/09/26.
18. UNFPA. Male Involvement in Reproductive Health Including Family Planning and Sexual Health. UNFPA Technical Report 28. . New York.1995.
19. Helzner JF. Men's Involvement in Family Planning. Reproductive Health Matters. 1996;4(7):146-54.
20. Homsy J, King R, Malamba S, Opio C, Kalamya JN, Mermin J, et al. The Need for Partner Consent Is a Main Reason for Opting Out of Routine HIV Testing for Prevention of Mother-to-Child Transmission in a Rural Ugandan Hospital. JAIDS. 2007;44(3):366-9.
21. Maman S, Moodley D, Groves A. Defining Male Support During and After Pregnancy from the perspective of HIV-Positive and HIV negative Women in Durban, South Africa. Journal of Midwifery and Women' s Hea;th. 2011;56(4):325-31.
22. Population Council. Research Update: Gender, Sexuality and HIV/AIDS. 2002.

23. De Cock KM, Fowler MG, Mercier E, de Vincenzi I, Saba J, Hoff E, et al. Prevention of Mother-to-Child HIV Transmission in Resource-Poor Countries, Translating Research Into Policy and Practice. JAMA. 2000;283(9):1175-82.
24. ANECCA. HIV/AIDS overview. African network for care of children affected by HIV/AIDS (ANNECA). USAID2008.
25. WHO. WHO. Towards universal access, scaling up priority HIV/AIDS interventions in the health sector, Progress report 2007. 2007 [cited 2012 14 April]. Available from: http://www.who.int/hiv/mediacentre/universal_access_progress_report_en.pdf
26. Nyasulu J. Factors Contributing to the Low Uptake of PMTCT Services in Blantyre and Balaka Rural.: University of Malawi, College of Medicine; 2007.
27. Bloom SS, Wypij D, Das GM. Dimensions of Women's autonomy and influence on maternal health care utilization in a north Indian City. Demography. 2001;38:67-78.
28. Beegle K, Frankenberg E, D T. Bargaining power within couples and use of prenatal and delivery care in Indonesia. 2001; 32:130-46. Stud Fam Plann. 2001;32:130-46.
29. Piet-Pelon N, Rob U, M.E K. Men in Bangladesh India and Pakistan: Reproductive Health Issues. . New Delhi : Population Council 1999.
30. Mlay R, Lugina H, Becker S. Couple counselling and testing for HIV at antenatal clinics: views from men, women and counsellors. AIDS Care. 2008;20(3):356-60. Epub 2008/03/21.
31. Baiden F, Remes P, Baiden R, Williams J, Hodgson A, M B. Voluntary Counselling and HIV testing for pregnant women in the Kassena-Nankana district of northern Ghana: Is couple counselling the way forward? AIDS Care. 2005;17(5):648-57.
32. Farquhar C, Mbori-Ngacha DA, Bosire RK, Nduati RW, Kreiss JK, GC J. Partner notification by HIV-1 seropositive pregnant women: association with infant feeding decisions. Aids. 2001;15(6):815-7.
33. Kiarie JN, Kreiss JK, Richardson BA, John-Stewart GC. Compliance with antiretroviral regimens to prevent perinatal HIV-1 transmission in Kenya. Aids. 2003;17(1):65-71. Epub 2002/12/13.
34. Msuya SE, Mbizvo EM, Hussain A, Uriyo J, Sam NE, B S-P. Low male partner participation in antenatal HIV counselling and testing in northern Tanzania: Implications for preventive programs. AIDS Care. 2008;20(6):700-9.
35. Gaillard P, Melis R, Mwanyumba F, Claeys P, Muigai E, Mandaliya K, et al. Vulnerability of women in an African setting: lessons for mother-to-child HIV transmission prevention programmes. Aids. 2002;16(6):937-9.
36. Akarro RRJ, Deonisia M, FJ. S. An Evaluation of Male Involvement on the Programme for PMTCT of HIV/AIDS: A Case Study of Ilala Municipality in Dar es Salaam, Tanzania. Arts and Social Sciences Journal. 2011;ASSJ-20 1.
37. Kebaabetswe PM. Barriers to participation in the prevention of mother-to-child HIV transmission program in Gaborone, Botswana a qualitative approach. AIDS Care. 2007;19(3):355-60. Epub 2007/04/25.
38. Farquhar C, Kiarie J, Richardson B, Kabura M, John F, Nduati R, et al. Antenatal Couple Counseling Increases Uptake of Interventions to Prevent HIV-1 Transmission. Journal of Acquired Immune Deficiency Syndrome 2004;37(5):1620-6.
39. Homsy J, Kalamya JN, Obonyo J, Ojwang J, Mugumya R, Opio C, et al. Routine intrapartum HIVcounseling and testing for prevention of mother-to-child transmission of HIV in a rural Ugandan hospital. Journal of Acquired Immune Deficiency Syndrome . 2006;42(2):149-54.
40. Alio AP, Salihu HM, Kornosky JL, Richman AM, Marty PJ. Feto-infant health and survival: does paternal involvement matter? Maternal and child health journal. 2010;14(6):931-7. Epub 2009/10/15.
41. Aarnio P, Olsson P, Chimbiri A, Kulmala T. Male involvement in antenatal HIV counseling and testing: exploring men's perceptions in rural Malawi. AIDS Care. 2009;21(12):1537-46. Epub 2009/12/22.
42. Semrau K, Kuhn L, Vwalika C, Kasonde P, Sinkala M, Kankasa C, et al. Women in couples antenatal HIV counseling and testing are not
more likely to report adverse social events. Aids. 2005 19(6):603-9.

43. Reece M, Hollub A, Nangami M, Lane K. Assessing male spousal engagement with prevention of mother-to-child transmission (pMTCT) programs in western Kenya. AIDS Care. 2010;22(6):743-50. Epub 2010/05/13.
44. Mullany BC. Barriers to and attitudes towards promoting husbands' involvement in maternal health in Katmandu, Nepal. Social science & medicine. 2006;62(11):2798-809. Epub 2005/12/27.
45. Mbuyi B, Matendo R, Vaz L, Callens S, Behets F, J K. Involving male partners in programs to prevent mother-to-child transmission of HIV in Kinshasa, DRC . Poster Exhibition: The XV International AIDS Conference: Abstract no. ThPeB7103" 2004. Available from: http://www.iasociety.org/Default.aspx?pageId=11&abstractId=2169317.
46. Falnes EF, Moland KM, Tylleskar T, de Paoli MM, Msuya SE, Engebretsen IM. "It is her responsibility": partner involvement in prevention of mother to child transmission of HIV programmes, northern Tanzania. Journal of the International AIDS Society. 2011;14(1):21. Epub 2011/04/28.
47. Burke M, Rajabu M, J. B. Maximising male participation in PMTCT programs in Tanzania. International AIDS Conference, July 11–16 2004; 15; abstract no. ThPeE8144.2004. Available from: http://www.iasociety.org/Default.aspx?pageId=11&abstractId=2175807.
48. Tweheyo R, Konde-Lule J, Tumwesigye NM, Sekandi JN. Male partner attendance of skilled antenatal care in peri-urban Gulu district, Northern Uganda. BMC pregnancy and childbirth. 2010;10:53. Epub 2010/09/18.
49. Byamugisha R, Tumwine JK, Semiyaga N, Tylleskar T. Determinants of male involvement in the prevention of mother-to-child transmission of HIV programme in Eastern Uganda: a cross-sectional survey. Reproductive health. 2010;7:12. Epub 2010/06/25.
50. Bwambale FM, Ssali SN, Byaruhanga S, Kalyango JN, Karamagi CA. Voluntary HIV counselling and testing among men in rural western Uganda: implications for HIV prevention. BMC public health. 2008;8:263. Epub 2008/07/31.
51. Nkuoh GN, Meyer DJ, Tih PM, Nkfusai J. Barriers to men's participation in antenatal and prevention of mother-to-child HIV transmission care in Cameroon, Africa. Journal of midwifery & women's health. 2010;55(4):363-9. Epub 2010/07/16.
52. Tshibumbu D. Factors Influencing Men's Involvement in Prevention of Mother to Child Transmission of HIV Programmes in Mambwe District, Zambia. South Africa: University Of South Africa; 2006.
53. Skovdal M, Campbell C, Nyamukapa C, Gregson S. When masculinity interferes with women's treatment of HIV infection: a qualitative study about adherence to antiretroviral therapy in Zimbabwe. Journal of the International AIDS Society. 2011;14:29. Epub 2011/06/11.
54. Mullany BC, Becker S, Hindin MJ. The impact of including husbands in antenatal health education services on maternal health practices in urban Nepal: results from a randomized controlled trial. Health education research. 2007;22(2):166-76. Epub 2006/07/21.
55. Theuring S, Mbezi P, Luvanda H, Jordan-Harder B, Kunz A, Harms G. Male involvement in PMTCT services in Mbeya Region, Tanzania. AIDS and behavior. 2009;13 Suppl 1:92-102. Epub 2009/03/25.
56. Amooti-Kaguna B, F. N. Factors influencing choice of delivery sites in Rakai district of Uganda. Social science & medicine. 2000;50(2):203-13.
57. Peacock D. Men as partners: Promoting men's involvement in care and support activities for people. Brazil:Engender Health: Brasilia; 2003.
58. Medley A, Garcia-Moreno C, McGill S, S M. Rates, barriers and outcomes of HIV serostatus disclosure among women in developing countries: implications for prevention of mother-to-child transmission programmes. Bulletin of the World Health Organization. 2004;82:299-307.
59. Martin LT, McNamara MJ, Milot AS, Halle T, Hair EC. The effects of father involvement during pregnancy on receipt of prenatal care and maternal smoking. Maternal and child health journal. 2007;11(6):595-602. Epub 2007/06/09.

60. Dev A. Involvement of husbands in the antenatal care: evaluation of Deepak Charitable Trust's outreach programme. Kathmandu gender workshop. In: Men as Supportive Partners in Reproductive and Sexual Health. Narrating Experiences. Kathmandu, Nepal: Population Council Workshop; 1998.
61. Bhalerao V R, Galwankar M, Kowli S S. Contribution of the education of the prospective fathers to the success of maternal health care programme. Journal of Postgraduate Medicine. 1984;10:10-2.
62. Brugha-Brugha R F, Kevany J P, Swan A V. An investigation of the role of fathers in immunization uptake. International Journal of Epidemeology 25: 840-5. 1996;25:840-5.
63. Khan M E. Involvig men in safe motherhood. Journal of Family Welfare. 1997;25:840-5.
64. Aluisio A, Richardson BA, Bosire R, John-Stewart G, Mbori-Ngacha D, Farquhar C. Male antenatal attendance and HIV testing are associated with decreased infant HIV infection and increased HIV-free survival. Journal of acquired immune deficiency syndromes. 2011;56(1):76-82. Epub 2010/11/19.
65. Wringe A, Isingo R, Urassa M, Maiseli G, Manyalla R, Changalucha J, et al. Uptake of HIV voluntary counselling and testing services in rural Tanzania: implications for effective HIV prevention and equitable access to treatment. Tropical medicine & international health : TM & IH. 2008;13(3):319-27. Epub 2008/04/10.
66. Sarker M. The Uptake of Prevention of mother to Child transmission (PMTCT) services in Nouna, Burkina Faso. 2006.
67. Mindry D, Maman S, Chirowodza A, Muravha T, van Rooyen H, Coates T. Looking to the future: South African men and women negotiating HIV risk and relationship intimacy. Culture, health & sexuality. 2011;13(5):589-602. Epub 2011/04/01.
68. Dudgeon MR, Inhorn MC. Men's influences on women's reproductive health: medical anthropological perspectives. Social science & medicine. 2004;59(7):1379-95. Epub 2004/07/13.
69. Sternberg P, Hubley J. Evaluating men's involvement as a strategy in sexual and reproductive health promotion. Health promotion international. 2004;19(3):389-96. Epub 2004/08/13.
70. Waweru M N K, Kiarie J N, Gathatwa FN, John-Stewart G. Interventions to increase male involvement in voluntary counseling and testing in antenatal care clinic. The XV International AIDS Conference Bangkok2004.
71. Mohlala BK, Boily MC, Gregson S. The forgotten half of the equation: randomized controlled trial of a male invitation to attend couple voluntary counselling and testing. Aids. 2011;25(12):1535-41. Epub 2011/05/26.
72. Jobson G. Changing masculinities: land-use, family communication and prospects for working with older men towards gender equality in a livelihoods intervention. Culture, health & sexuality. 2010;12(3):233-46. Epub 2009/11/12.
73. Pool R, Nyanzi S, Whitworth JA. Attitudes to voluntary counselling and testing for HIV among pregnant women in rural south-west Uganda. AIDS Care. 2001;13(5):605-15. Epub 2001/09/26.
74. Katz DA, Kiarie JN, John-Stewart GC, Richardson BA, John FN, Farquhar C. HIV testing men in the antenatal setting: understanding male non-disclosure. International journal of STD & AIDS. 2009;20(11):765-7. Epub 2009/10/17.
75. Larsson EC, Thorson A, Pariyo G, Conrad P, Arinaitwe M, Kemigisa M, et al. Opt-out HIV testing during antenatal care: experiences of pregnant women in rural Uganda. Health policy and planning. 2012;27(1):69-75. Epub 2011/02/05.
76. Msellati P. Improving mothers' access to PMTCT programs in West Africa: A public health perspective. Social science & medicine. 2009;69(6):807-12.
77. Kululanga LI, Sundby J, Malata A, Chirwa E. Male Involvement in Maternity Health Care in Malawi. African Journal of Reproductive Health. 2012;16(1):145-58.
78. O'Gorman DA, Nyirenda LJ, Theobald SJ. Prevention of mother-to-child transmission of HIV infection: views and perceptions about swallowing nevirapine in rural Lilongwe, Malawi. BMC public health. 2010;10:354. Epub 2010/06/23.
79. Tadesse E, Muula AS, H. M. Likely stakeholders in the prevention of mother to child transmission of HIV/AIDS in Blantyre, Malawi

African Health Sciences. 2004;4(3):155-9.
80. Nyasulu J, Nyasulu P. Decision making for women to access prevention of mother to child transmission services in Blantyre and Balaka Districts, Malawi. Journal of Rural and Tropical Public Health. 2011;10:95-100.
81. Kasenga F. Making it happen: prevention of mother to child transmission of HIV in rural Malawi. Global health action. 2010;3. Epub 2010/07/08.
82. Muheriwa SR. Factors Influencing Utilization of Prevention of Mother to Child Transmission of HIV Services in Young Adults in Balaka District. Lilongwe: University of Malawi, Kamuzu College of Nursing; 2011.
83. van Lettow M, Bedell R, Landes M, Gawa L, Gatto S, Mayuni I, et al. Uptake and outcomes of a prevention-of mother-to-child transmission (PMTCT) program in Zomba district, Malawi. BMC public health. 2011;11:426. Epub 2011/06/07.
84. Masingi N, Chabwera C, Chauwa F, Kandikole L, Jumbe A, Nkhalamba T, et al. Experience with couples attending voluntary counseling and testing in Lilongwe, Malawi. The XV International AIDS Conference; Bangkok2004.
85. Rankin SH, Lindgren T, Rankin WW, Ng'Oma J. Donkey work: women, religion, and HIV/AIDS in Malawi. Health care for women international. 2005;26(1):4-16. Epub 2005/03/15.
86. Kululanga LI, Sundby J, Malata A, Chirwa E. Striving to promote male involvement in maternal health care in rural and urban settings in Malawi - a qualitative study. Reproductive health. 2011;8:36. Epub 2011/12/03.
87. Greene L, Mercer S. Precede-Proceed Model. Encyclopedia of Public Health [Internet]. 2002. Available from: http://www.encyclopedia.com/doc/1G2-3404000676.html.
88. Glanz K, Rimer B, K V, editors. Health Behavior and Health Education: Theory, Research, and Practice. Edition 4 ed: Wiley, John & Sons, Incorporated September 2008
89. Hulley SB, Cummings SR. Designing Clinical Research, An Epidemiologic Approach. Baltimore: Williams & Wilkins; 1998.
90. NSO. Population and Housing Census Results. . Zomba: National Statistical Office; 2008. p. 1-23.
91. Byamugisha R, Astrom AN, Ndeezi G, Karamagi CA, Tylleskar T, Tumwine JK. Male partner antenatal attendance and HIV testing in eastern Uganda: a randomized facility-based intervention trial. Journal of the International AIDS Society. 2011;14:43. Epub 2011/09/15.
92. Polit D, Hungler B. Nursing Research, Principles and Methods. Philadelphia: Lippincott; 1999.
93. Neuman WL. Social Research Methods Qualitative and Quantitative Approaches. USA: Pearson Inc; 2006.
